# The Urinary Transcriptome as a Source of Biomarkers for Prostate Cancer

**DOI:** 10.3390/cancers12020513

**Published:** 2020-02-22

**Authors:** Carla Solé, Ibai Goicoechea, Alai Goñi, Maike Schramm, María Armesto, María Arestin, Lorea Manterola, Maitena Tellaetxe, Aitor Alberdi, Leonor Nogueira, Mathieu Roumiguie, Jose Ignacio López, Juan Pablo Sanz Jaka, Ander Urruticoechea, Itziar Vergara, Ana Loizaga-Iriarte, Miguel Unda, Arkaitz Carracedo, Bernard Malavaud, Charles H. Lawrie

**Affiliations:** 1 Molecular Oncology group, Biodonostia Research Institute, 20014 San Sebastián, Spain; carla.sole@biodonostia.org (C.S.); ibai.goicoechea@biodonostia.org (I.G.); m.schramm02@googlemail.com (M.S.); maria.armesto@biodonostia.org (M.A.); maria.arestin@biodonostia.org (M.A.); lorea.manterola@biodonostia.org (L.M.); maitena.tellaeche@biodonostia.org (M.T.); 2 Multiple Myeloma Group, Centro de Investigación Médica Aplicada (CIMA), Pamplona, 31008 Navarra, Spain; 3 Radiation Oncology, Onkologikoa Foundation, 20014 Gipuzkoa, Spain; agoni@onkologikoa.org; 4 Faculty of Biosciences, University of Heidelberg, 69047 Heidelberg, Germany; 5 Hernani Primary Care Health Center, OSI Donostialdea, 20120 Osakidetza, Spain; aitor.alberdiazcue@osakidetza.eus; 6 Laboratory of Cell Biology and Cytology, Purpan University Hospital, 31059 Toulouse, France; nogueira.l@chu-toulouse.fr; 7 Department of Urology, Institut Universitaire du Cancer, 31100 Toulouse, France; roumiguie.mathieu@iuct-oncopole.fr (M.R.); bernard.malavaud@me.com (B.M.); 8 Department of Pathology, Cruces University Hospital, 48903 Barakaldo, Spain; joseignacio.lopez@osakidetza.eus; 9 Biomarkers in Cancer Unit, Biocruces Research Institute, 48903 Barakaldo, Spain; 10 Department of Medical-Surgical Specialties, University of the Basque Country (UPV/EHU), 48940 Leioa, Spain; 11 Department of Urology, Hospital Donostia, 20014 San Sebastián, Spain; juanpablo.sanzjaka@osakidetza.eus; 12 Onkologikoa Foundation, 20014 Gipuzkoa, Spain; anderu@onkologikoa.org; 13 Unidad de Investigación AP OSIs Gipuzkoa, Biodonostia Research Institute, 20014 San Sebastián, Spain; itziar.vergara@biodonostia.org; 14 Red de Investigación en Servicios de Salud en Enfermedades Crónicas (REDISSEC), 48903 Barakaldo, Spain; 15 Department of Urology, Basurto University Hospital, 48013 Bilbao, Spain; ana.loizagairiarte@osakidetza.net (A.L.-I.); jesusmiguel.undaurzaiz@osakidetza.net (M.U.); 16 CIBERONC (Centro de Investigación Biomédica en Red de Cáncer), 28020 Madrid, Spain; acarracedo@cicbiogune.es; 17 Department of Biochemistry and Molecular Biology, University of the Basque Country (UPV/EHU), 48940 Leioa, Spain; 18 CIC bioGUNE, Bizkaia Technology Park, Building 801a, 48160 Derio, Spain; 19 IKERBASQUE, Basque Foundation for Science, 48005 Bilbao, Spain; 20 Radcliffe Department of Medicine, University of Oxford, Oxford pOX13 9DU, UK

**Keywords:** prostate cancer, PSA, liquid biopsy, biomarker, mRNA, transcriptome, NGS

## Abstract

Prostate cancer (PCa) is the second most common cancer of men and is typically slow-growing and asymptomatic. The use of blood PSA as a screening method has greatly improved PCa diagnosis, but high levels of false positives has raised much interest in alternative biomarkers. We used next-generation sequencing (NGS) to elucidate the urinary transcriptome of whole urine collected from high-stage and low-stage PCa patients as well as from patients with the confounding diagnosis of benign hyperplasia (BPH). We identified and validated five differentially expressed protein-coding genes (*FTH1 BRPF1*, *OSBP*, *PHC3*, and *UACA*) in an independent validation cohort of small-volume (1 mL) centrifuged urine (*n* = 94) and non-centrifuged urine (*n* = 84) by droplet digital (dd)PCR. These biomarkers were able to discriminate between BPH and PCa patients and healthy controls using either centrifuged or non-centrifuged whole urine samples, suggesting that the urinary transcriptome is a valuable source of non-invasive biomarkers for PCa that warrants further investigation.

## 1. Introduction

Prostate cancer (PCa) is the second most common cancer in men worldwide and the fifth leading cause of cancer mortality in this gender [1]. Typically, PCa is a slow-growing tumor, and up to 40% of diagnosed men present without any clinical symptoms at all. However, patients have a five-year survival rate >99% if PCa is diagnosed at an early stage (i.e., localized) but only a ~30% survival rate if it is diagnosed at an advanced, high-grade metastatic stage [1]. As a consequence, early diagnostic PCa screening has the potential to radically improve the healthcare burden of this cancer.

The most widely used screening test for PCa is the detection of elevated serum levels of prostate-specific antigen (PSA) or of the closely related (−2) proPSA [2,3,4]. However, this test is far from perfect as even moderately increased PSA levels (i.e., ≥4 ng/mL) can be associated with cofounding diagnoses such as benign hyperplasia (BPH) or prostatic inflammation [5]. Indeed, in men with PSA levels between 4 to 10 ng/mL, this biomarker has a specificity of only 20–40%, which leads many of them to undergo an unnecessary prostate biopsy, a costly, invasive, and uncomfortable procedure [6]. Furthermore, over a quarter of men who are diagnosed with PCa have normal PSA levels (i.e., ≤4 mg/mL) [7]. Therefore, there is a clear clinical need for alternative non-invasive biomarkers for this cancer.

Urine is a particularly attractive source of biomarkers for PCa screening due to the ease of testing when compared to blood and may capture the disease at an earlier stage. Several studies have demonstrated that urine contains many different species of RNA, including mRNA [8], microRNAs (miRNAs), and other non-coding (nc)RNAs [9]. Indeed, aside from PSA blood tests, the only other FDA-approved tests for PCa are the detection of the long non-coding RNA (lncRNA) PCA3 in urine [10,11] and, more recently, the SelectMDx test, whose diagnostic performance remains to be clearly established [12]. Whilst several studies have investigated miRNAs in the urine of PCa patients [13,14,15], the biomarker potential of other RNA species have yet to be fully explored. We therefore used RNAseq to elucidate the entire circulating urinary transcriptomes of low-stage (LS) PCa patients, high-stage (HS) PCa patients, and patients with the confounding diagnosis of BPH. These findings were validated in a cohort of 178 urine samples, including a comparison of centrifuged urine (CU) samples (*n* = 94) and non-centrifuged urine (NCU) samples (*n* = 84).

## 2. Results

### 2.1. Sequencing the Circulating Transcriptome of Urine from Prostate Cancer Patients

Due to the low quantity of cfRNA obtained from urine (average yield 45 ng/mL of urine), we pooled samples from five individuals according to whether they were diagnosed with BPH, LS PCa (i.e., stage I/II), or HS PCa (i.e., stage IIII/IV), as depicted in Table 1. Individual patient details are given in Table S1. Purified total RNA were used to build libraries, and the libraries underwent next-generation sequencing (NGS) using an Illumina HiSeq sequencer as described in Materials (Section 3).

We obtained an average of 11.9 million reads per sample (range 11.3–12.7), with a minimum of 81% of reads passing quality control (QC) filtering (Phred score >20) (Table S4). Between 51% and 75% of reads were mapped to the human genome (HS19), and 46,964 different annotated transcripts were detected in the samples (>10 reads per transcript). Of these, there were 316 distinct miRNAs detected in the samples, which accounted for 2.2%, 1.2%, and 2.7% of the total mapped reads in the BPH, LS, and HS samples respectively. The most commonly expressed miRNAs were miR-663a, miR-663b, miR-1977, miR-30c, miR-181d, and miR-29c.

The top 50 transcripts (by average read count) accounted for 35%, 16%, and 29% of the total read counts in BPH, LS, and HS samples, respectively (Table S5). This list contained mainly mRNA protein-coding transcripts (37/50), with a large proportion of mitochondrially encoded transcripts (15/50) and five members of the MUC (mucin) family. There were 13 ncRNA transcripts, including 4 members of the signal recognition particle RNA (RNA7SL), 2 long non-coding RNAs (AC010970.2 and AC079949.1), 1 miRNA (miR-663), 1 YRNA (RNY4), and 1 snoRNA (snoU13).

By pair-wise comparisons, we identified 327 transcripts that were differentially expressed (adjusted *p* < 0.05) in BPH and LS samples, of which 140 were down-regulated, and 187 were up-regulated in LS samples (Table S6). There were 63 transcripts differentially expressed in BPH and HS samples, with 29 down-regulated in HS samples and 34 up-regulated (Table S7), and 341 differentially expressed transcripts in LS and HS samples, with 158 up-regulated in HS samples and 183 down-regulated (Table S8).

**Figure 1:**
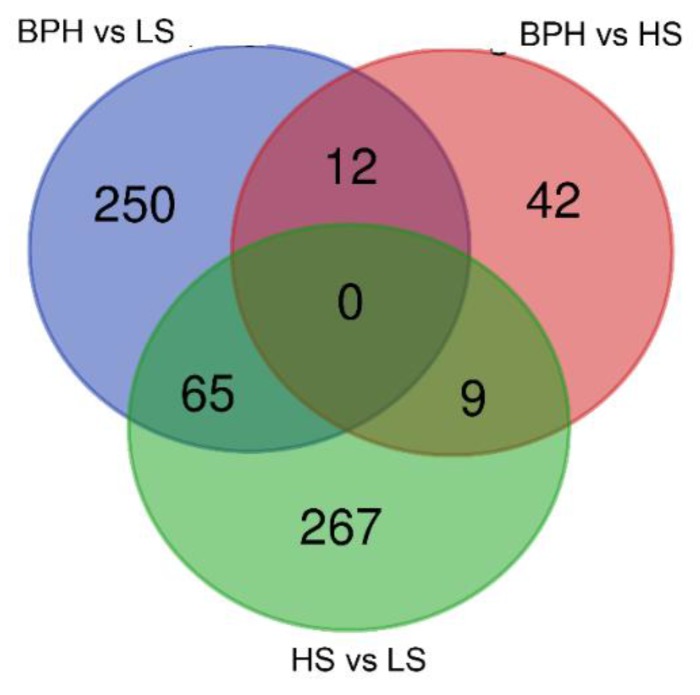
Venn diagram of differentially expressed genes showing overlap between different groupings.

There were 12 transcripts in common between (BPH vs. LS) and (BPH vs. HS), 9 common transcripts between (BPH vs. HS) and (LS vs. HS), and 65 common transcripts between (BPH vs. LS) and (LS vs. HS) (Figure 1 and Tables S6–S8).

Ontology pathway analysis of the differentially expressed transcripts between BPH and LS (*n* = 327), BPH and HS (*n* = 63), and HS and LS (*n* = 341) showed a significant enrichment for 12, 4, and 21 pathways, respectively (Supplementary Tables S9–S11). Of note, RAN signaling pathways were the first and fourth most significant pathways in HS vs. LS samples and BPH vs. HS samples, respectively. In addition, the protein ubiquitination pathway was the 2nd and 13th most significant pathway in BPH vs. LS and HS vs. LS samples, respectively. The molecules involved in the protein ubiquitination pathway for each of these data sets were non-overlapping (Supplementary Figure S1).

### 2.2. Validation in Centrifuged and Non-Centrifuged Urine Cohorts

**Figure 2:**
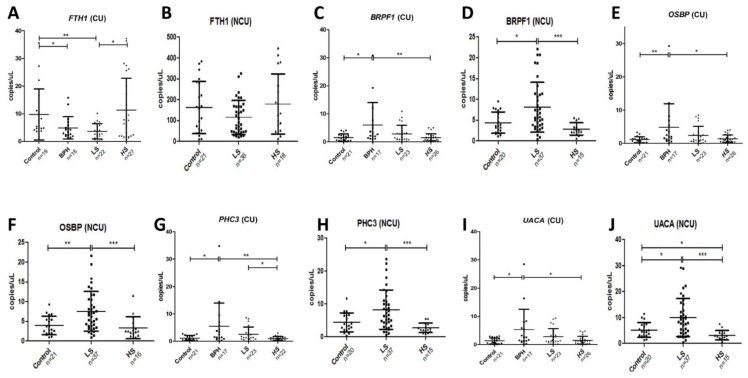
Expression levels of mRNA (copies/μL) measured by droplet digital (dd)PCR in independent validation cohorts of CU and NCU samples. Expression levels were compared using Mann–Whitney independent t-test (* *p* ≤ 0.05; ** *p* ≤ 0.01; *** *p* ≤ 0.001). LS, low stage (stage I and II); HS, high stage (stage III and IV). (**A**) Levels of *FTH1* in CU samples. (**B**) Levels of *FTH1* in NCU samples (**C**) Levels of *BRPF1* in CU samples (**D**) Levels of *BRPF1* in NCU samples (**E**) Levels of *OSBP* in CU samples (**F**) Levels of *OSBP* in NCU samples. (**G**) Levels of *PHC3* in CU samples. (**H**) Levels of *PHC3* in NCU samples. (**I**) Levels of *UACA* in CU samples. (**J**) Levels of *UACA* in NCU samples.

Five differentially expressed gene fragments. i.e., ferritinheavy chain 1 (FTH1), bromodomain and PHD finger-containing protein 1 (BRPF1), oxysterol-binding protein 1 (OSBP), polyhomeotic-like protein 3 (PHC3), and uveal autoantigen with coiled-coil domains and ankyrin repeats (UACA), were selected for further investigation on the basis of being either consistently up-regulated (FTH1) or consistently down-regulated (BRPF1, OSBP, PHC3, and UACA) in the pooled NGS samples. Taqman probes were designed (Table S12) to detect these targets, and their levels were measured by droplet digital (dd)PCR in individual samples (non-pooled samples) of CU collected from 73 PCa patients and 21 age-matched healthy controls, in addition to individual NCU samples from 60 PCa patients and 24 age-matched healthy controls (Table 1; Figure 2).

Consistent with the NGS data, we observed that the levels of FTH1 were significantly higher in CU-HS samples compared to CU-BPH samples, and in the latter they were higher than in CU-LS samples (Figure 2A). Surprisingly, CU samples from healthy control volunteers also displayed high levels of FTH1, which were significantly higher than the levels in either CU-BPH or CU-LS samples and were similar to the levels in CU-HS samples (9.75 cf. 11.37, respectively). The pattern of FTH1 expression in NCU samples were similar to those of CU samples between the groups, but the variability of the levels within these groups meant that differences were not statistically significant (Figure 2B). In contrast to the other genes measured in this study, there was a big increase in the levels of FTH1 measured in NCU compared to CU samples, with an average (NCU/CU) ratio of 15.2 (112.2/7.4) compared to rations of 1.98, 2.3, 2.32, and 2.63 for BRPF1, OSBP, PHC3, and UACA, respectively. Again, consistent with the NGS data, the levels of BRPF1 in CU-BPH samples were higher than those in either CU-LS or CU-HS (and CU-control) samples, although in the former group of samples they were not significantly lower than in CU-BPH samples (Figure 2C). In NCU samples, a similar pattern was again observed compared to CU samples; however, in NCU-LS samples, the levels of BRPF1were also significantly higher than in NCU-HS samples (Figure 2D). The levels of OSBP were also higher in CU-BPH samples compared to CU-HS and CU-control samples but not compared to CU-LS samples (Figure 2E). Similarly to BRPH1, the levels of OSBP in NCU samples mirrored those of CU samples, with the exception of NCU-LS samples that displayed much higher levels of OSBP than CU-LS samples (Figure 2F). The levels of PHC3 were higher in CU-BPH samples compared to either CU-control or CU-HS samples (Figure 2G). In addition, the levels of PHC3 were significantly higher in CU-LS samples compared to CU-HS samples. The NCU samples also displayed the same pattern as the CU samples in regard to PHC3 expression (Figure 2H). The levels of UACA were also higher in CU-BPH samples compared to other CU samples (Figure 2I); they were also higher in NCU samples, although similarly high levels were observed in NCU-LS samples (Figure 2J).

To investigate the biomarker potential of the gene fragments in the urine samples, we carried out a receiver operator curve (ROC) analysis comparing the ability of the individual genes to discriminate between PCa and/or BPH samples and control samples (Table 2). We also combined the genes in a panel using the Panelomix algorithm [16]. The best preforming genes to discriminate between BPH and control CU samples were PHC3 and FTH1, with an area-under-curve AUC value of 0.782. The same genes were also shown be the best discriminators between CU-BPH samples and CU-HS or CU-PCa (i.e., LS and HS samples), with AUC values of 0.771 and 0.711, respectively. Interestingly, although FTH1 was not always the gene with the highest AUC value in the ROC analysis, it was always included in an optimized panel design, even in NCU samples.

**Figure 3:**
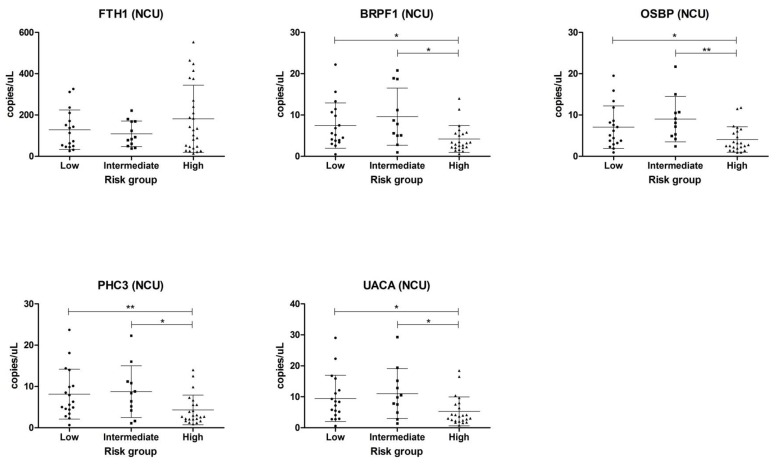
Expression levels of mRNA (copies/μL) in NCU samples classified according to risk groups in accordance with to the D’Amico score. Expression levels were compared using Mann–Whitney independent t-test (* *p* ≤ 0.05; ** *p* ≤ 0.01).

To study these genes as predictive biomarkers, we additionally analyzed their expression between low-, intermediate-, and high-risk groups according to the D’Amico score. We only analyzed NCU samples, as the CU cohort patients were all defined as high-risk. In NCU samples, the levels of BRPF1, OSBP, PHC,3 and UACA showed significant differences when comparing high-risk, low-risk, and intermediate-risk patients (Figure 3). In addition, the genes were analyzed by classifying patients according to the Gleason score In NCU samples, we failed to find any significant differences between Gleason groups (Supplemental Figure S2) but did find differences between Gleason 7 (intermediate-aggressiveness) and Gleason ≥8 (high-aggressiveness) cases for BRPF1 levels. In contrast, the levels of OSBP and PHC3 were significantly different between Gleason ≤6 (poor aggressiveness) and Gleason ≥8 (Supplemental Figure S3) cases.

## 3. Discussion

Urine represents an attractive source of biomarkers for urogenital cancers, and its potential ease of collection makes it an ideal candidate for widespread screening. In particular, the need for a viable alternative to PSA testing for PCa screening has generated a great interest in urine as a source of biomarkers in recent years. Compared to blood, the urinary circulating transcriptome has been little studied, and the vast majority of studies have been carried out on miRNAs and, specifically, on their presence in urine extracellular vesicles (uEVs) [15,17,18,19,20,21,22]. Even though uEVs could represent an enriched source of biomarkers for PCa diagnosis, the need for highly specialized purification instrumentation such as ultracentrifuges makes such technique beyond the reach of the vast majority of routine clinical diagnostic laboratories. We used NGS to elucidate the urinary transcriptome of high-stage and low-stage PCa patients in comparison with that of patients with the confounding diagnosis of BPH, which is a frequent cause of false positives in PSA testing, leading to unnecessary, expensive, and uncomfortable surgical diagnostic interventions. The results of our study and other studies suggest that miRNAs represent only a small proportion of the RNA species present in whole urine, with the majority of RNA being ribosomal RNA (rRNA) and mitochondrial-associated RNA [23]. Interestingly, mitochondrial RNAs have previously been identified as potential urinary biomarkers in bladder cancer [24]. We identified 645 differentially expressed transcripts in a pair-wise comparison, the vast majority of which were protein-encoding. We did not identify either *PCA3* or *PSA* mRNA (the basis of the PCA3 test) or *DLX1* and *HOXC6* mRNA (the basis of the SelectMDx test) as, although expressed, they did not reach statistical significance in our analyses (data not shown). Pathway analysis of the differentially expressed genes showed common enrichment for genes in protein ubiquitination and RAN signaling pathways. RAN is a GTPase belonging to the RAS family, which is frequently dysregulated in both androgen-dependent [25] and androgen-independent PCa [26]. The role of protein ubiquitination in the development and progression of PCa is well characterized [27], and indeed the ubiquitination-targeting drug Bortezomib has been the subject of several clinical trials for PCa [28].

We validated five candidate mRNA biomarkers (*FTH1*, *BRPF1*, *OSBP*, *PHC3*, and *UACA*) in 94 centrifuged and 84 non-centrifuged small-volume (1 mL) whole-urine samples. As far as we are aware, this is the first study to investigate coding gene transcript biomarkers derived from whole urine. *BRPF1* is a core subunit of the histone acetyltransferase complex that acts as a tumor suppressor in childhood leukemia and adult medulloblastoma [29] and an oncogene in acute myeloid leukemia (AML) [30]. *BRPF1* interacts with the MOZ and MORF proteins, the latter being frequently mutated in castration-resistant PCa [31]. *OSBP* and OSBP-related proteins (ORPs) constitute a large family of proteins responsible for protein transport and regulate sterols and phospholipids metabolism [32]. Members of this family are frequently de-regulated in cancer, including ORP5 in pancreatic cancer and lung cancer [33,34], ORP7 in breast cancer [35], and OSBP2 in cholangiocarcinoma [36]. *PHC3* is a member of the polycomb complex involved in epigenetic programming, whose downregulation has been correlated with poor prognostic outcome in PCa [37]. *UACA* has previously been described to be upregulated in PCa and is involved in the regulation of apoptosis as a target of p53 [38,39].

Even though the urine samples used in the NGS cohort were obtained after prostatic massage, we were able to validate the genes without prostatic massage in both centrifuged and non-centrifuged urine. Furthermore, in general, there was little difference in the patterns of expression of the biomarkers between CU and NCU samples, at least for *BRPF1*, *OSBP*, *PHC3*, and *UACA*, suggesting that these transcripts were not cellular-associated but cell-free in the urine. A notable exception was *FTH1*, whose levels were >15-fold higher in NCU samples, suggesting it was associated with cells, although not necessarily tumor cells, as it was present in comparable quantities in both healthy control and BPH urine samples. *FTH1* has recently been identified as a key regulator of tumorigenesis in PCa in association with *FTH1* pseudogene and *miR-638* [40]. Consistent with our data, these authors found that levels of *FTH1* were downregulated in PCa. We extended these findings to urine samples and showed that *FTH1* levels decreased in BPH samples and were the lowest in LS urine samples. However, we found that the levels were higher in HS samples than in LS samples, both in CU and NCU samples, perhaps suggesting that other mechanisms are responsible for controlling iron concentration in metastatic PCa patients. We were unable to detect *miR-638* in any of the urine samples tested in this study (data not shown). In contrast to *FTH1*, we observed the highest levels of *BRPF1*, *OSBP*, *PHC3*, and *UACA* in samples taken from patients with BPH, with levels of all four genes being significantly higher in CU samples compared to healthy control samples or PCa-HS samples, although not necessarily PCa-LS samples. The same genes where highly expressed in LS NCU samples compared with samples from healthy and HS patients. The ability to differentiate between BPH and PCa patients, which often present overlapping PSA levels and similar symptoms, could reduce the number of unnecessary surgical interventions and also could help to take therapeutic decisions. In our study, *UACA* levels in NCU were able to differentiate non-cancerous patients from PCa patients (LS and HS). Although *BRPF1*, *OSBP*, and *PHC3* levels in NCU samples could only distinguish LS patients from non-cancerous and HS patients, it is speculated that they could be used as a biomarker to identify the initial steps of the disease and, therefore, as a guide to apply local therapy. Moreover, as the genes in NCU samples could distinguish between high-risk patients (D’Amico risk score) and low- and intermediate-risk patients, they could be used to inform therapeutic decisions.

## 4. Materials and Methods

### 4.1. Patient Selection

The samples used in this study were collected from multiple centers both retrospectively (*n* = 58) and prospectively (*n* = 128). For the NGS cohort, urine was collected prospectively after prostate massage from 15 BPH and PCa patients attending the Hopitaux de Toulouse, Toulouse, France (Table 1 and Table S1). For the validation cohorts, samples were obtained without prostatic massage retrospectively from the Basque Biobank for research O + EHUN (CEIC code OHEUN11–12 and OHEUN14–14) (*n* = 58) and prospectively from Onkologikoa Cancer Hospital (*n* = 75). The samples were collected from patients at the time of diagnosis prior to any treatment and were collected as first urine of the day (between 8 AM and 10 AM) under fasting conditions. Urine from age-matched healthy controls (*n* = 38) were obtained from the Basque Biobank for Research O + EHUN. For CU samples, urine was centrifuged at 3000 g for 5 min at room temperature. Written informed consent was obtained from patients for the inclusion of their samples in this study, and the samples were collected in accordance with the Declaration of Helsinki and with approval by local ethics committees (CEIC-Euskadi approval number PI2015076).

### 4.2. RNA Purification and Library Construction

Individual donor samples (20 mL volume each) were pooled according to disease stage, with five samples per pool (i.e., 100 mL total urine), as shown in Table S1. Total RNA was purified using the Urine RNA Concentration-Preservation and Isolation Kit from Norgen Biotek (Ontario, Canada). Average yields were 45 ng mL^−1^ of urine. In total, 3 µg of RNA was used for each library. Ribosomal RNA (rRNA) was removed from total cfRNA using the Ribozero Magnetic Human/Mouse/Rat kit (Illumina, #MRZH116, San Diego, CA 92122), and Truseq libraries were prepared using 20 cycles of PCR of barcoded primers. Sequencing was performed on an Illumina HiSeq 400 using 50 PE in rapid mode.

### 4.3. Bioinformatic Analysis

Sequencing reads were QC-filtered and adapter-trimmed using the Fastq Groomer aLSorithm that forms part of the Galaxy suite of programs. Reads were mapped to the GRCh37 build of the human genome using the Bowtie 2.0 aLSorithm. MiRNA expression was calculated using the miraligner aLSorithm from the seqBuster suite, and YRNA expression was calculated using the HTseq-count algorithm. Differential expression analysis was carried out using the DESeq bioconductor package [41]. Ontology analysis was carried out using Ingenuity Pathway Analysis software from Qiagen (Germantown, MD, USA).

### 4.4. Droplet Digital PCR (ddPCR)

For validation of mRNAs, mRNA from non-pooled samples was reverse-transcribed (RT) using random primers with the High-Capacity cDNA Reverse Transcription Kit from Applied Biosystems, following the manufacturers’ protocol. Due to the difficulty in quantifying cfRNA reliably, we used fixed volumes in the reactions [42,43]. For the mRNA fragments detected, we designed custom Taqman probes using the Custom TaqMan^®^ Assay Design Tool from Applied Biosystems. ddPCR was performed using QX200 TM Droplet DigitalTM PCR system (Bio-Rad), following the manufacturers’ protocol. Data analysis was performed by QuantaSoft analysis software from Bio-Rad. Expression levels were compared using Mann–Whitney independent *t*-test (Graphpad Prism v. 5.0, La Jolla, CA, USA). ROC analysis and comparisons were carried out using the method of DeLong et al., as implemented in MedCalc v. 14.8 software [63].

## 5. Conclusions

In summary, through the elucidation of the whole urinary transcriptome of BPH and PCa patients, we identified and independently validated five mRNA biomarkers detectable by ddRT-PCR in 1 mL samples obtained from either centrifuged or non-centrifuged urine. Whilst it is clear that further validation is required, this study suggests that urine is a valuable source of biomarkers that surely merits further exploration.

## Figures and Tables

**Table 1:** Summary of different cohorts used for this study.

Sample Type	Stage	*N*	Age (Median)	Average PSA
**NGS cohort**	BPH	5	68	4.4
	Low stage (LS)	5	70	6.7
	High stage (HS)	5	68	16.1
**Centrifuged urine (CU)**	Control	21	69	-
	BPH	21	72	-
	LS	25	64	NK
	HS	27	69	NK
**Non-centrifuged urine (NCU)**	Control	24	67	-
	LS	41	69	8.49
	HS	19	70	21.31
Total	-	193	-	-

Details of individual patients in the discovery cohort and centrifuged and non-centrifuged validation cohorts can be found in Tables S1–S3, respectively. NGS, next-generation sequencing: NK, not known; BPH, benign hyperplasia; LS, low stage (stage I and II); HS, high stage (stage III and IV); PSA, prostate-specific antigen.

**Table 2:** ROC analysis of selected mRNA biomarkers.

Probe		Cont.*	*(CU)*			BPH *	*(CU)*			Cont + BPH *	*(CU)*	Cont.*	*(NCU)*
	BPH	LS	HS	LS + HS	LS	HS	LS + HS	LS	HS	LS + HS	LS	HS	LS + HS
*FTH1*	0.715	0.773	0.506	0.626	0.773	0.618	0.523	0.654	0.510	0.579	0.557	0.515	0.534
*BRPF1*	0.741	0.596	0.551	0.518	0.642	0.768	0.709	0.521	0.648	0.568	0.641	0.577	0.572
*OSBP*	0.741	0.597	0.526	0.559	0.619	0.727	0.676	0.503	0.575	0.553	0.673	0.559	0.599
*PHC3*	0.720	0.637	0.554	0.543	0.601	0.761	0.679	0.513	0.567	0.543	0.649	0.580	0.576
*UACA*	0.713	0.651	0.509	0.576	0.611	0.727	0.668	0.553	0.597	0.552	0.648	0.616	0.565
Panel †	0.782 (*PHC3/FTH1*)	0.661 (*OSBP/FTH1*)	0.832 (*OSBP/FTH1/BRPF1*)	0.661 (*OSBP/FTH1*)	0.773 (*FTH1*)	0.771 (*PHC3/FTH1*)	0.711 *(PHC3/FTH1*)	0.654 (*FTH1*)	0.618 (*OSBP/FTH1*)	0.612 (*BRPF1/FTH1/UACA*)	0.605 (*OSBP/FTH1*)	0.621 (UACA/FTH1)	0.638 (*OSBP/FTH1*)

* Reference samples used as controls for ROC analysis. † Composition of panel genes given in parenthesis.

## Supplementary Materials

The following are available online at https://www.mdpi.com/2072-6694/12/2/513/s1, Figure S1: Genes that are differentially expressed in LS vs HS (top) and BPH vs LS (bottom) in the context of the protein ubiquitination pathway, Figure S2: Expression levels of selected genes in non-centrifuged urine samples (NCU), classified according to Gleason score, Figure S3: Expression levels of selected genes in centrifuged urine samples, Table S1: Details of patients used for NGS, Table S2: Clinical details of individual patients and healthy controls used in the centrifuged urine (CU) validation cohort, Table S3: Clinical details of individual patients and healthy controls used in the non-centrifuged (NCU) urine validation cohort, Table S4: Summary of NGS results from pooled RNA samples, Table S5: Top 50 most abundant transcripts by average read count normalized by transcript length, Table S6: Differentially expressed (*p* < 0.05) transcripts in BPH vs. LS samples, Table S7: Differentially expressed (*p* < 0.05) transcripts in BPH vs. HS samples, Table S8: Differentially expressed (*p* < 0.05) transcripts in HS vs. LS samples, Table S9: Statistically significant pathways for differentially expressed transcripts in BPH and LS samples (*n* = 327), Table S10: Statistically significant pathways for differentially expressed transcripts in BPH and HS samples (*n* = 63), Table S11: Statistically significant pathways for differentially expressed transcripts in LS and HS samples (*n* = 341), Table S12: Sequences used for Taqman probe design.
