# Supplementary material for: The Urinary Transcriptome as a Source of Biomarkers for Prostate Cancer
Source: Cancers (Basel). 2020 Feb 22;12(2):513. doi: 10.3390/cancers12020513 (PMC7072374; doi:10.3390/cancers12020513)
Supplement: Supplementary file 1 [file cancers-12-00513-s001.pdf]

cancers

MDPI

Supplementary Materials

Table S1. Details of patients used for NGS. BPH, benign prostate hyperplasia; LS, low stage (stage I and II); HS, high stage (stage III and IV).

Sample ID	Age	Gleason	PSA	pT	pN	pM	Stage	PCA3	RNA	Pool
2163	79	<5	6.50	-	-	pM0	-	35	69	BPH
1045	74	<5	0.27	-	-	pM0	-	30	35	BPH
1103	68	<5	4.10	-	-	pM0	-	34	48	BPH
1237	59	<5	4.45	-	-	pM0	-	14	38	BPH
2124	64	<5	6.94	-	-	pM0	-	32	41	BPH
1082	77	6	8.74	cT1a	pN0	pM0	IIA	44	24	LS
2039	71	6	4.17	pT2c	pN0	pM0	IIB	47	36	LS
2074	65	8	7.23	pT2c	pNx	pM0	IIB	24	34	LS
1272	63	7	3.17	pT2c	pNx	pM0	IIB	67	33	LS
2066	74	6	9.93	cT2c	pN0	pM0	IIA	60	33	LS
1145	61	9	12.37	pT3b	pN1	pM0	IV	51	63	HS
1200	70	8	1.86	pT3a	pN1	pM0	IV	nd	87	HS
1256	68	8	5.22	pT3a	pN0	pM0	III	73	35	HS
2002	67	7	26.46	pT3a	pN0	pM0	III	23	37	HS
2047	74	7	34.18	pT3a	pNx	pM0	III	26	47	HS

Table S2. Clinical details of individual patients and healthy controls of the centrifuged urine (CU) validation cohort.

Sample ID	Age	Gleason	PSA	Tumor (pT)	Lymph nodes (pN)	Metastasis (pM)	Stage
AC436	68	-	-	BPH	-	pM0	BPH
AC392	69	-	-	BPH	-	pM0	BPH
AC382	83	-	-	BPH	-	pM0	BPH
AC400	70	-	-	BPH	-	pM0	BPH
AC406	57	-	-	BPH	-	pM0	BPH
AC489	76	-	-	BPH	-	pM0	BPH
AC415	82	-	-	BPH	-	pM0	BPH
AC421	72	-	-	BPH	-	pM0	BPH
AC425	64	-	-	BPH	-	pM0	BPH
AC430	62	-	-	BPH	-	pM0	BPH
AC433	70	-	-	BPH	-	pM0	BPH
AC444	76	-	-	BPH	-	pM0	BPH
AC435	77	-	-	BPH	-	pM0	BPH
AC440	75	-	-	BPH	-	pM0	BPH
AC443	86	-	-	BPH	-	pM0	BPH
AC462	69	-	-	BPH	-	pM0	BPH
AC449	70	-	-	BPH	-	pM0	BPH
AC451	76	-	-	BPH	-	pM0	BPH
AC457	68	-	-	BPH	-	pM0	BPH
AC486	61	-	-	BPH	-	pM0	BPH
AC416	80	-	-	BPH	-	pM0	BPH
AC434	60	6		pT2a	pNx	pM0	Stage IIA
AC453	68	7		pT2c	pNx	pM0	Stage IIB
AC459	53	7		pT2c	pNx	pM0	Stage IIB
AC465	62	7		pT2c	pNx	pM0	Stage IIB
AC483	63	7		pT2c	pNx	pM0	Stage IIB
AC480	62	9		pT2a	pNx	pM0	Stage IIB
AC490	50	7		pT2c	pNx	pM0	Stage IIB
AC495	69	7		pT2c	pNx	pM0	Stage IIB
AC500	66	7		pT2c	pNx	pM0	Stage IIB
AC391	69	7		pT2c	-	pM0	Stage IIB
AC432	68	8		pT2c	pNx	pM0	Stage IIB
AC438	57	6		pT2c	pNx	pM0	Stage IIB

AC390V2	67	8		pT2c	pN0	pM0	Stage IIB
AC442	66	7		pT2c	pNx	pM0	Stage IIB
AC447	53	7		pT2c	pN0	pM0	Stage IIB
AC454	71	7		pT2c	pNx	pM0	Stage IIB
AC502	69	6		pT2c	pNx	pM0	Stage IIB
AC393	55	6		pT2c	pNx	pM0	Stage IIB
AC405	71	7		pT2c	pNx	pM0	Stage IIB
AC446	74	8		pT2c	pNx	pM0	Stage IIB
AC496	64	6		pT2c	pNx	pM0	Stage IIB
AC419	72	7		pT2c	pNx	pM0	Stage IIB
AC455	59	7		pT2c	pNx	pM0	Stage IIB
AC498	60	7		pT2c	pNx	pM0	Stage IIB
GARONK-003	77	9	11	T2a	pN0	pM0	Stage IIB
AC460	72	7		pT3a	pNx	pM0	Stage III
AC479	55	8		pT3a	pN0	pM0	Stage III
AC485	56	7		pT3a	pN0	pM0	Stage III
AC441	66	7		pT3a	pNx	pM0	Stage III
AC456	72	7		pT3a	pNx	pM0	Stage III
AC461	64	7		pT3a	pNx	pM0	Stage III
AC481	72	9		pT3b	pN0	pM0	Stage III
AC452	68	7		pT3a	pNx	pM0	Stage III
AC492	61	8		pT3a	pNx	pM0	Stage III
AC450	73	7		pT3a	pNx	pM0	Stage III
GARONK-001	70	6	5.69	T3a	pN0	pM0	Stage III
GARONK-002	70	9	6.94	T3b	pN0	pM0	Stage III
GARONK-006	73	7	3.44	T3a	pN0	pM0	Stage III
GARONK-008	77	9	15	T3a	pN0	pM0	Stage III
GARONK-009	66	8	6.85	T3a	pN0	pM0	Stage III
GARONK-012	74	6	7	T3a	pN0	pM0	Stage III
GARONK-013	78	7	4.9	T3a	pN0	pM0	Stage III
GARONK-017	63	9	7.7	T3b	pN0	pM0	Stage III
GARONK-018	72	9	6	T3b	pN0	pM0	Stage III
GARONK-019	75	7	4.8	T3a	pN0	pM0	Stage III
GARONK-011	81	9	16.5	T2c	pN0	pM0	Stage III
GARONK-014	71	7	13.9	T3b	pN0	pM0	Stage III
GARONK-015	64	6	3	T3a	pN0	pM0	Stage III
GARONK-016	79	7	6	T3a	pN0	pM0	Stage III
AC493	61	9		pT3b	pN1	pM0	Stage IV
AC505	61	8		pT3b	pN1	pM0	Stage IV
AC494	65	8		pT3a	pN1	pM0	Stage IV
30619	75	-	-	-	-	-	Control
30640	81	-	-	-	-	-	Control
30576	70	-	-	-	-	-	Control
30656	71	-	-	-	-	-	Control
30706	75	-	-	-	-	-	Control
30682	72	-	-	-	-	-	Control
30689	72	-	-	-	-	-	Control
30667	71	-	-	-	-	-	Control
30692	84	-	-	-	-	-	Control
30708	73	-	-	-	-	-	Control
30810	73	-	-	-	-	-	Control
HER_001	65	-	-	-	-	-	Control
HER_002	41	-	-	-	-	-	Control
HER_003	64	-	-	-	-	-	Control
HER_004	76	-	-	-	-	-	Control
HER_005	59	-	-	-	-	-	Control
HER_006	66	-	-	-	-	-	Control
HER_007	55	-	-	-	-	-	Control

HER_008	61	-	-	-	-	-	Control
HER_009	76	-	-	-	-	-	Control
HER_010	63	-	-	-	-	-	Control

Table S3. Clinical details of individual patients and healthy controls of the non-centrifuged (NCU) urine validation cohort.

Sample ID	Age	Gleason	PSA	Tumour (pT)	Lymph nodes (pN)	Metastasis (pM)	Stage
ONK-003	74	6	8.6	cT2a	pN0	pM0	Stage I
ONK-022	68	6	8.8	cT1c	pN0	pM0	Stage I
ONK-029	59	6	3.7	cT2a	pN0	pM0	Stage I
ONK-040	60	6	5.4	cT1c	pN0	pM0	Stage I
ONK-052	77	6	9.97	cT2a	pN0	pM0	Stage I
ONK-054	71	6	6.7	cT1c	pN0	pM0	Stage I
ONK-059	65	6	2.72	cT1c	pN0	pM0	Stage I
ONK-062	63	6	8.4	cT1c	pN0	pM0	Stage I
ONK-063	74	6	6	cT2a	pN0	pM0	Stage I
ONK-069	51	6	9.4	cT2a	pN0	pM0	Stage I
ONK-071	70	6	6.57	cT2a	pN0	pM0	Stage I
ONK-074	68	6	4.6	cT2a	pN0	pM0	Stage I
ONK-075	75	6	10	cT1c	pN0	pM0	Stage I
ONK-044	74	6	8.8	cT2a	pN0	pM0	Stage I
ONK-067	71	6	7.7	cT2a	pN0	pM0	Stage I
ONK-083	79	6	7	cT1c	pN0	pM0	Stage I
ONK-025	68	6	6.56	cT2a	pN0	pM0	Stage I
ONK-010	78	6	9.3	cT2a	pN0	pM0	Stage I
ONK-002	73	6	8.45	cT2a	pN0	pM0	Stage I
ONK-039	74	6	5.4	T2a	pN0	pM0	Stage I
ONK-041	72	6	7.6	T1a	pN0	pM0	Stage I
ONK-011	80	7	9.3	cT1c	pN0	pM0	Stage IIA
ONK-013	78	7	5	cT2a	pN0	pM0	Stage IIA
ONK-016	73	7	19.43	cT2a	pN0	pM0	Stage IIA
ONK-017	75	6	6	T2b	pN0	pM0	Stage IIA
ONK-024	63	6	7.14	cT2b	pN0	pM0	Stage IIA
ONK-028	63	7	5.5	cT2b	pN0	pM0	Stage IIA
ONK-037	71	6	19	cT2a	pN0	pM0	Stage IIA
ONK-065	72	6	15	cT2a	pN0	pM0	Stage IIA
ONK-076	67	7	10	cT2a	pN0	pM0	Stage IIA
ONK-082	63	7	8	cT2a	pN0	pM0	Stage IIA
ONK-068	69	6	5.7	T2b	pN0	pM0	Stage IIA
ONK-19-05	77	6	6.3	T2b	pN0	pM0	Stage IIA
ONK-009	68	6	6	cT2c	pN0	pM0	Stage IIB
ONK-027	64	6	22	cT2b	pN0	pM0	Stage IIB
ONK-046	51	6	8.14	cT2c	pN0	pM0	Stage IIB
ONK-061	62	7	X	cT2c	pN0	pM0	Stage IIB
ONK-084	72	7	6.35	cT2c	pN0	pM0	Stage IIB
ONK-004	63	4	2.8	cT2c	pN0	pM0	Stage IIB
ONK-057	79	8	20.87	T2b	pN0	pM0	Stage IIB
ONK-19-04	78	8	3.9	T2c	pN0	pM0	Stage IIB
ONK-006	68	7	16.48	cT3b	pN0	pM0	Stage III
ONK-015	62	7	34	cT3b	pN0	pM0	Stage III
ONK-019	62	8	87	cT3b	pN0	pM0	Stage III
ONK-023	74	6	12.8	cT3a	pN0	pM0	Stage III
ONK-026	68	8	6.8	cT3a	pN0	pM0	Stage III
ONK-034	73	8	8.45	cT3b	pN0	pM0	Stage III
ONK-035	72	8	16.29	cT3a	pN0	pM0	Stage III
ONK-042	70	6	12.88	cT3a	pN0	pM0	Stage III

ONK-072	60	6	5.24	cT3a	pN0	pM0	Stage III
ONK-078	75	6	8	cT3a	pN0	pM0	Stage III
ONK-19-01	74	7	7.3	T3b	pN0	pM0	Stage III
ONK-19-02	73	7	6.7	T3a	pN0	pM0	Stage III
ONK-19-03	71	6	10	T3a	pN0	pM0	Stage III
ONK-005	69	6	12.18	cT3a	pN0	pM0	Stage III
ONK-007	68	8	61.8	cT3b	pN0	pM0	Stage III
ONK-038	76	7	7.41	T3b	pN0	pM0	Stage III
ONK-001	64	6	5.66	cT3a	pN0	pM0	Stage III
ONK-19-06	80	8	79.3	T3b	pN0	pM0	Stage III
ONK-19-07	73	7	6.6	T3a	pN0	pM0	Stage III
HER_001	65	-	-	-	-	-	Control
HER_002	41	-	-	-	-	-	Control
HER_003	64	-	-	-	-	-	Control
HER_004	76	-	-	-	-	-	Control
HER_005	59	-	-	-	-	-	Control
HER_006	66	-	-	-	-	-	Control
HER_007	55	-	-	-	-	-	Control
CV001	63	-	-	-	-	-	Control
CV002	69	-	-	-	-	-	Control
CV003	70	-	-	-	-	-	Control
CV004	77	-	-	-	-	-	Control
CV005	66	-	-	-	-	-	Control
CV006	72	-	-	-	-	-	Control
CV008	72	-	-	-	-	-	Control
CV009	63	-	-	-	-	-	Control
CV013	68	-	-	-	-	-	Control
CV014	74	-	-	-	-	-	Control
CV015	70	-	-	-	-	-	Control
CV016	70	-	-	-	-	-	Control
CV017	73	-	-	-	-	-	Control
30656 NC	71	-	-	-	-	-	Control
CV010	66	-	-	-	-	-	Control
CV011	75	-	-	-	-	-	Control
CV012	72	-	-	-	-	-	Control

Table S4. Summary of NGS results from pooled RNA samples. *Average % of reads with Phred score >20.

Pool	*Reads (10^6)*	*QC**	*Mapped (10^6) (%)*
BPH	11.6	81.7	7.07 (60.7)
LS PCa	11.3	89.8	8.49 (75.3)
HS PCa	12.7	81.8	6.48 (51.1)

Table S5. Top 50 most abundant transcripts by average read count normalized by transcript length. BPH, benign prostate hyperplasia; LS, low stage (stage I and II); HS, high stage (stage III and IV).

	**BPH**	**LS**	**HS**
AL161626.1 (rRNA_pseudogene 17)	1291565	277046	492569
CTD-2328D6.1 (RNA28S5)	585838	8833	815881
AC079949.1 (lncRNA)	447029	17369	476421
AC010970.2 (lncRNA)	75625	4125	81389
MT-ND5	52206	229273	28468
MT-ND6	49973	179344	26499
MT-ND2	22924	110066	13266
MT-ND4	16137	134277	10725
MT-CYB	13321	108790	4884
ROCK1P1	12991	4433	9493
RN7SL1	12078	1188	8756

RN7SL2	10758	1342	5709
MT-CO1	9185	51931	2750
MT-ATP6	9042	83226	4774
MT-ND1	8558	62964	3454
MT-RNR2	7359	78694	8921
RNA5-8SP6	6039	1518	16720
Y-RNA3	5863	14201	5720
MT-CO2	4301	30976	1287
TTN	3993	7975	3586
MUC12	3982	6952	4147
MT-RNR1	3861	12958	1265
MIR663A	3729	924	3927
(BRPF1*)	3674	1560	605
RP11-91I20.3	3630	3817	1485
MT-CO3	3344	14927	858
RN7SL4P	3300	286	3058
KCNQ1OT1	3212	8921	2816
RN7SL5P	3212	198	3740
MUC16	3201	3641	2981
MUC3A	2827	9031	6501
MTND2P28	2354	12815	770
REXO1L1P	2310	3036	1650
HYDIN	2288	1925	2398
CDC27P1	2068	3773	2585
NBPF1	1991	3806	2607
MUC4	1969	4565	3322
MT-ND3	1947	18403	1199
snoU13	1936	4400	2266
(PHC3*)	1903	3663	2200
OBSCN	1870	891	1870
CDC27	1837	1804	1221
MICAL3	1837	891	2068
MUC6	1837	3982	2112
MTATP6P1	1815	3036	660
SSPO	1782	550	704
SYNE1	1760	2706	1221
RP11-413E6.7	1749	440	1540
COL6A3	1727	2409	1155

Table 6. Differentially expressed ($P < 0.05$) transcripts in BPH and LS samples**.** Genes depicted in bold type are also present in the LS vs. HS list (Supplementary Table S6). Genes depicted in bold type and underlined are also present in the BPH vs. HS list (Supplementary Table S5).

Genes	BPH	LS	Fold Change	adj. *P*-value
MTND1P23	350	11858	33.89	4.69E-04
<u>***EEF1A1P6***</u>	1575	76	-20.66	1.24E-03
PRRC1	97	1722	17.72	1.53E-03
(FTH1*)	1844	117	-15.81	1.61E-03
EFR3A	136	1692	12.43	4.04E-03
EIF3F	136	1692	12.43	4.15E-03
MLIP	175	1844	10.54	4.34E-03
RP11-96K19.4	233	2241	9.61	4.56E-03
CENPE	117	1463	12.55	4.77E-03
CBWD6	253	2362	9.35	4.80E-03
ANKRD36	253	2271	8.99	4.95E-03
CDK18	1147	76	-15.05	4.97E-03
<u>***SECISBP2L***</u>	97	1128	11.60	5.73E-03
STXBP5	97	1341	13.80	6.05E-03
FOXP2	253	2103	8.32	6.78E-03

<u>DICER1</u>	117	1311	11.24	6.80E-03
MTND5P7	117	1448	12.41	6.87E-03
EEF1A1	1575	168	-9.39	8.08E-03
LDLR	1108	107	-10.39	8.14E-03
DHRS2	1069	91	-11.69	8.20E-03
XRN1	214	1844	8.62	8.64E-03
PPFIA2	97	1204	12.39	8.70E-03
CNTLN	253	1997	7.90	8.81E-03
TMEM45A	136	1357	9.97	8.84E-03
HAP1	953	76	-12.50	8.86E-03
FAM126B	136	1433	10.53	8.98E-03
CD55	97	1128	11.60	9.19E-03
GOLGA4	311	2271	7.30	9.61E-03
EIF4G3	272	2012	7.39	9.78E-03
MT-ATP8	991	10319	10.41	9.78E-03
DYNC2H1	330	2378	7.20	9.81E-03
TCAIM	136	1402	10.31	9.97E-03
KTN1	428	2926	6.84	1.06E-02
SEMA3A	214	1783	8.34	1.08E-02
GABRA2	233	1814	7.78	1.10E-02
<u>CTDSP2</u>	991	91	-10.84	1.11E-02
FIP1L1	97	1082	11.13	1.11E-02
CCDC144NL	156	1402	9.02	1.13E-02
ARHGEF38	97	1097	11.29	1.15E-02
(OSBP)*	5539	585	-9.47	1.20E-02
C8orf34	156	1402	9.02	1.22E-02
(PHC3)*	1903	122	-15.59	1.24E-02
ATAD5	214	1738	8.13	1.31E-02
ADAMTS14	836	76	-10.97	1.31E-02
<u>ZMAT1</u>	97	975	10.04	1.34E-02
TPH1	117	1174	10.06	1.35E-02
ATAD1	97	1036	10.66	1.36E-02
TRIP11	175	1402	8.02	1.37E-02
LUC7L3	156	1311	8.43	1.38E-02
PSMG3-AS1	816	76	-10.71	1.40E-02
MYO5BP2	97	1021	10.51	1.41E-02
SMC5	97	1006	10.35	1.43E-02
TAOK1	253	1799	7.12	1.45E-02
TMEM86A*	719	76	-9.44	1.46E-02
PABPC1	97	1006	10.35	1.48E-02
MYT1	1069	122	-8.77	1.48E-02
PLCH2	797	76	-10.46	1.51E-02
LHCGR	97	975	10.04	1.54E-02
MIER1	136	1235	9.07	1.56E-02
PLEKHA8	175	1402	8.02	1.56E-02
STX7	272	1783	6.55	1.56E-02
TMCO5A	136	1265	9.30	1.57E-02
BTAF1	175	1387	7.93	1.57E-02
ANKRD18B	97	975	10.04	1.62E-02
SCFD1	97	975	10.04	1.62E-02
ANKS1A	1186	137	-8.64	1.63E-02
MTND5P27	156	1296	8.33	1.63E-02
PSMD6	175	1417	8.10	1.64E-02
RP11-848G14.5	875	91	-9.57	1.67E-02
GATAD2A	739	76	-9.69	1.67E-02
<u>RPL37</u>	136	1052	7.73	1.70E-02
COL9A3	758	76	-9.95	1.72E-02
SPATA6L	175	1296	7.41	1.73E-02
F7	680	76	-8.93	1.73E-02
HNRNPD	175	1402	8.02	1.74E-02
MITD1	97	930	9.57	1.75E-02
PTCHD2	816	91	-8.93	1.77E-02

PRPF4B	156	1311	8.43	1.82E-02
PTAR1	156	1296	8.33	1.84E-02
EDC4	739	76	-20.66	1.88E-02
NAALAD2	97	899	9.25	1.90E-02
SQSTM1	1575	244	-6.46	1.94E-02
PAPD4	156	1280	8.23	1.96E-02
FAM47E	175	1326	7.58	1.98E-02
RALGAPA1	233	1494	6.40	1.99E-02
EEF2	1497	229	0.15	2.02E-02
MSRB3	97	899	9.25	2.03E-02
GOLGA8T	700	76	-9.18	2.09E-02
ZNF638	467	2637	5.65	2.10E-02
SLC37A2	953	122	-7.81	2.11E-02
STXBP5*L*	136	1143	8.40	2.11E-02
GALC	175	1250	7.14	2.13E-02
C14orf164	136	1113	8.18	2.14E-02
DNAJB11	117	1006	8.62	2.15E-02
CCDC144CP	136	1128	8.29	2.17E-02
DNAH14	428	2378	5.56	2.18E-02
SLC25A29	894	107	-8.38	2.20E-02
ZCCHC11	544	3018	5.54	2.24E-02
MTND5P6	97	869	8.94	2.24E-02
HSPBAP1	97	869	8.94	2.24E-02
KIRREL*3*	1225	198	-6.18	2.29E-02
ABI1	97	854	8.78	2.30E-02
COL18A1	991	137	-7.23	2.34E-02
ARFGAP1	622	76	-8.16	2.34E-02
NLE1	680	76	-8.93	2.35E-02
PATE4	97	823	8.47	2.36E-02
CTC-228N24.3	117	975	8.36	2.37E-02
RAB21	136	1097	8.06	2.38E-02
PFKL	641	76	-8.42	2.44E-02
METTL14	136	1082	7.95	2.45E-02
C1orf222	1244	183	-6.80	2.45E-02
TONSL	758	91	-8.29	2.45E-02
FASN	797	107	-7.47	2.46E-02
FBXW7	117	960	8.23	2.47E-02
PKD1	1886	351	-5.38	2.47E-02
ANKRD27	1613	259	-6.23	2.50E-02
AZIN1	156	1128	7.25	2.51E-02
TNS3	1438	259	-5.55	2.53E-02
PARVG	1225	198	-6.18	2.54E-02
U2SURP	311	1814	5.83	2.55E-02
ANAPC15	622	76	-8.16	2.56E-02
CAMK2D	117	945	8.10	2.58E-02
TMEM116	97	823	8.47	2.60E-02
CHFR	1400	229	-6.12	2.60E-02
SMAD1	117	930	7.97	2.60E-02
RCOR3	233	1417	6.08	2.60E-02
KCNQ2	836	107	-7.83	2.63E-02
TCP11L2	136	1006	7.39	2.65E-02
PRKCI	136	1052	7.73	2.67E-02
PLEKHA3	408	2149	5.26	2.68E-02
MMAA	97	808	8.31	2.71E-02
FRS2	136	1036	7.62	2.72E-02
ANKRD12	311	1768	5.68	2.72E-02
MAP3K19	97	808	8.31	2.73E-02
LY6E	603	76	-7.91	2.75E-02
APLNR	564	76	-7.40	2.78E-02
AGAP3	1516	274	-5.53	2.78E-02
SMARCD3	816	107	-7.65	2.80E-02
DHX29	97	777	8.00	2.80E-02

RP11-1281K21.6	97	777	8.00	2.83E-02
TEX9	292	1738	5.96	2.83E-02
ANKRD36	641	3231	5.04	2.83E-02
ENGASE	816	107	-7.65	2.85E-02
SCARA5	505	76	-6.63	2.85E-02
PTEN	97	777	8.00	2.86E-02
FER1L5	1769	320	-5.53	2.87E-02
FLT4	1069	183	-5.85	2.87E-02
CAPS2	136	1021	7.50	2.88E-02
SMARCA4	797	122	-6.54	2.89E-02
BAAT	97	747	7.68	2.89E-02
ICAM1	525	76	-6.89	2.89E-02
YAP1	175	1189	6.80	2.90E-02
KIRREL	816	122	-6.70	2.91E-02
HSPA4L	175	1174	6.71	2.94E-02
NT5DC2	700	91	-7.65	2.94E-02
ERBB2IP	233	1494	6.40	2.94E-02
ZC3H7B	1283	213	-6.01	2.94E-02
NEK8	622	76	-8.16	2.95E-02
POU3F3	117	899	7.71	2.95E-02
USP19	894	122	-7.33	2.96E-02
CRHR1	680	91	-7.44	2.98E-02
DNAJC2	233	1433	6.14	3.00E-02
RSPH3	97	732	7.53	3.02E-02
MKLN1	136	1006	7.39	3.03E-02
SLC12A2	136	1006	7.39	3.03E-02
DSCAML1	1108	183	-6.06	3.03E-02
ESRP1	97	716	7.37	3.04E-02
RP11-958N24.1	525	76	-6.89	3.04E-02
SNAPC4	525	76	-6.89	3.04E-02
RP11-112N13.1	117	884	7.58	3.04E-02
RP11-461G12.2	117	884	7.58	3.05E-02
DPCR1	544	2713	4.98	3.05E-02
C1orf216	641	91	-7.01	3.07E-02
VRK2	97	747	7.68	3.07E-02
SNN	505	76	-6.63	3.07E-02
RP11-597A11.1	97	732	7.53	3.08E-02
BDP1	350	1814	5.18	3.09E-02
IRX4	272	76	-3.57	3.14E-02
JOSD1	564	76	-7.40	3.14E-02
PAPOLA	311	1692	5.44	3.14E-02
SPTLC3	311	1753	5.64	3.15E-02
POT1-AS1	117	869	7.45	3.16E-02
C9orf153	97	701	7.21	3.17E-02
HNRNPK	175	1204	6.88	3.17E-02
OR11J2P	292	76	-3.83	3.19E-02
RP11-973H7.1	292	76	-3.83	3.19E-02
VWA2	428	76	-5.61	3.19E-02
KCNIP3	1225	213	-5.74	3.21E-02
PRSS8	603	76	-7.91	3.22E-02
CHRND	311	76	-4.08	3.24E-02
ZNF765	953	137	-6.94	3.24E-02
ITPKB	855	122	-7.01	3.24E-02
RHPN1	661	91	-7.23	3.24E-02
HGS	661	91	-7.23	3.29E-02
MIR3916	350	76	-4.59	3.30E-02
MISP	350	76	-4.59	3.30E-02
RP11-344P13.3	350	76	-4.59	3.30E-02
SRP68	855	122	-7.01	3.32E-02
CYB5R4	233	1448	6.21	3.33E-02
ELFN2	1089	168	-6.49	3.35E-02
MTND4P27	97	747	7.68	3.35E-02

RP11-383H13.1	97	747	7.68	3.35E-02
WDR43	136	930	6.83	3.37E-02
ART3	97	701	7.21	3.38E-02
ANKRD29	117	854	7.32	3.40E-02
UGP2	97	747	7.68	3.40E-02
ANXA9	641	91	-7.01	3.41E-02
NTRK1	661	91	-7.23	3.42E-02
SPATA17	97	732	7.53	3.42E-02
TDO2	117	808	6.93	3.45E-02
RP6-42F4.1	136	945	6.94	3.46E-02
ZBTB46	758	107	-7.11	3.50E-02
RP11-347C12.2	117	838	7.19	3.51E-02
GHDC	447	76	-5.87	3.52E-02
UFL1	117	838	7.19	3.53E-02
ZNF844	739	107	-6.92	3.54E-02
RP11-15B24.5	117	838	7.19	3.55E-02
TBCB	505	76	-6.63	3.55E-02
CYP4F12	544	76	-7.14	3.55E-02
TAF5L	97	671	6.90	3.56E-02
GRK1	914	137	-6.66	3.58E-02
RB1	253	1417	5.61	3.61E-02
ATG3	97	655	6.74	3.64E-02
PCYT2	428	76	-5.61	3.65E-02
CDIP1	914	137	-6.66	3.65E-02
ULK1	1089	183	-5.95	3.67E-02
ZBED3-AS1	117	777	6.66	3.70E-02
WDR7	253	1311	5.19	3.72E-02
RAB3D	894	137	-6.52	3.72E-02
L1CAM	661	91	-7.23	3.72E-02
PYGO1	117	823	7.06	3.73E-02
PAPD5	136	930	6.83	3.74E-02
MT-ND4L	661	3018	4.57	3.76E-02
TP53TG3D	97	701	7.21	3.76E-02
RPL3P4	505	76	-6.63	3.76E-02
SLC15A3	739	107	-6.92	3.77E-02
CUEDC1	739	107	-6.92	3.78E-02
RNF38	156	975	6.27	3.80E-02
ACAP3	816	122	-6.70	3.82E-02
NEDD4	350	1768	5.05	3.83E-02
LACC1	175	1128	6.45	3.84E-02
FAM78A	603	91	-6.59	3.85E-02
ZBTB21	97	701	7.21	3.88E-02
C19orf25	700	107	-6.56	3.88E-02
SVIP	97	655	6.74	3.90E-02
EMID1	719	107	-6.74	3.92E-02
PRPF38A	156	960	6.17	3.92E-02
ZNF213	641	91	-7.01	3.96E-02
USP15	175	1021	5.84	3.98E-02
NCOA3	350	1738	4.97	4.01E-02
SMCHD1	525	2393	4.56	4.02E-02
MTND6P9	97	625	6.43	4.03E-02
RERGL	97	625	6.43	4.03E-02
RFX6	97	625	6.43	4.03E-02
RRN3P1	97	625	6.43	4.03E-02
RIOK3	97	686	7.06	4.09E-02
DUX4L3	797	122	-6.54	4.10E-02
NPTX1	700	107	-6.56	4.10E-02
LSM5	97	625	6.43	4.12E-02
PTPLAD2	272	1448	5.32	4.13E-02
BLK	758	122	-6.22	4.14E-02
KDR	233	1235	5.29	4.14E-02
ATF3	564	91	-6.16	4.18E-02

ASB14	214	1280	5.99	4.21E-02
KCNG2	330	76	-4.34	4.21E-02
TTLL9	525	76	-6.89	4.22E-02
CTD-2281E23.1	486	76	-6.38	4.23E-02
CYP2D8P	700	107	-6.56	4.23E-02
ADCY7	1089	183	-5.95	4.23E-02
CCDC14	272	1433	5.26	4.25E-02
DECR1	97	686	7.06	4.26E-02
FAF1	97	686	7.06	4.26E-02
SH3GLB2	544	91	-5.95	4.31E-02
MBOAT2	214	1250	5.84	4.33E-02
WDR5	855	137	-6.24	4.34E-02
KIF15	156	975	6.27	4.38E-02
SH3RF3	700	107	-6.56	4.38E-02
RNPEPL1	700	107	-6.56	4.38E-02
(BRPF1*)	3674	944	-3.95	4.40E-02
PTCD1	292	76	-3.83	4.43E-02
CYHR1	758	122	-6.22	4.45E-02
ATP13A1	505	76	-6.63	4.46E-02
AKR1A1	447	76	-5.87	4.51E-02
PRKCD	467	76	-6.12	4.51E-02
ABCC13	447	2012	4.50	4.52E-02
GPR98	875	3887	4.44	4.53E-02
FBRSL1	1147	229	-5.10	4.53E-02
RMDN3	680	107	-6.38	4.53E-02
CORO2B	505	91	-5.53	4.55E-02
EHD1	836	137	-6.09	4.57E-02
A1BG	272	76	-3.57	4.57E-02
ACSL5	136	854	6.27	4.58E-02
GUF1	97	640	6.59	4.59E-02
TANC2	525	2317	4.41	4.60E-02
HAX1	505	76	-6.63	4.61E-02
TBC1D31	136	838	6.16	4.61E-02
FLNC	1264	259	-4.88	4.63E-02
RP11-846F4.5	233	1250	5.36	4.64E-02
DNM1P47	1302	274	-4.75	4.67E-02
ST6GALNAC6	622	107	-5.83	4.67E-02
(UACA)*	991	183	-5.42	4.70E-02
SESN3	156	945	6.08	4.72E-02
TCF25	680	107	-6.38	4.72E-02
PGBD5	544	91	-5.95	4.72E-02
COPB1	156	945	6.08	4.74E-02
TXNDC16	156	945	6.08	4.74E-02
DCPS	505	76	-6.63	4.74E-02
TDRD7	156	945	6.08	4.76E-02
ABCB4	330	1494	4.52	4.76E-02
CNPY4	253	76	-3.32	4.79E-02
NBEA	253	1311	5.19	4.79E-02
SEZ6	486	76	-6.38	4.80E-02
YME1L1	156	930	5.98	4.81E-02
ZC3HC1	117	701	6.01	4.82E-02
EARS2	564	91	-6.16	4.82E-02
RP11-115N12.1	97	625	6.43	4.85E-02
RCOR1	117	686	5.88	4.85E-02
LRP1B	408	1783	4.37	4.87E-02
RSPH9	350	76	-4.59	4.90E-02
DYRK1B	233	76	-3.06	4.91E-02
PHF5A	233	76	-3.06	4.91E-02
CD3G	136	808	5.94	4.91E-02
LDLRAP1	739	122	-6.06	4.94E-02
FBXO44	447	76	-5.87	4.94E-02
TBX1	739	122	-6.06	4.96E-02

ADCY9	1380	290	-4.77	4.99E-02
TMEM184A	739	122	-6.06	5.00E-02

Table S7. Differentially expressed (*P*<0.05) transcripts in BPH vs. HS samples. Genes depicted in bold type and underlined are also present in the BPH vs. LS list (Supp. Table S4). Genes depicted in red are also present in the LS vs. HS list (Supp. Table S6).

Genes	BPH	HS	Fold Change	adj. *P*-value
SECISBP2L	97	1325	13.63	3.17E-03
RPL37	136	1287	9.46	8.58E-03
RANBP1	97	1037	10.67	1.29E-02
AKAP8	97	999	10.27	1.45E-02
ZBED3-AS1	117	1056	9.06	1.48E-02
RP13-279N23.2	117	1095	9.38	1.62E-02
SETSIP	175	1402	8.01	1.83E-02
HNRNPA3P12	136	1056	7.76	2.09E-02
ZMAT1	97	807	8.30	2.34E-02
PDZD7	816	96	-8.50	2.34E-02
CLIC5	156	1133	7.29	2.50E-02
SV2C	816	96	-8.50	2.53E-02
COPS5	97	826	8.50	2.54E-02
FAM84A	97	807	8.30	2.73E-02
ADAMTS16	97	787	8.10	2.75E-02
(UACA)*	991	101	-7.49	2.76E-02
PRCD	1127	154	-7.34	2.81E-02
ANKRD27	1613	269	-6.00	2.87E-02
CTDSP2	991	134	-7.38	2.90E-02
METAP1	97	787	8.10	2.91E-02
NNT	758	96	-7.90	2.97E-02
SLC6A2	136	999	7.34	3.03E-02
PKLR	1089	154	-7.09	3.11E-02
PRDM11	855	115	-7.42	3.14E-02
RASGRP4	719	96	-7.49	3.18E-02
DENND2D	156	1075	6.92	3.31E-02
FAM118A	214	1210	5.66	3.38E-02
MTND5P11	603	2996	4.97	3.39E-02
TCEA3	816	115	-7.09	3.53E-02
F11	97	730	7.51	3.58E-02
ZDHHC17	117	826	7.08	3.59E-02
SAFB	719	96	-7.49	3.62E-02
PFKFB4	700	96	-7.29	3.70E-02
DECR1	97	711	7.31	3.85E-02
FAM177A1	700	96	-7.29	3.90E-02
CHIA	797	115	-6.92	3.91E-02
ZNF45	117	807	6.92	3.92E-02
POLD4	622	96	0.15	4.04E-02
EEF1A1P6	1575	288	-5.47	4.05E-02
RP11-576I22.2	97	672	6.92	4.15E-02
AP2A2	1069	173	-6.19	4.15E-02
DNTTIP2	641	96	-6.68	4.16E-02
POLR1C	136	864	6.35	4.20E-02
WDR7	253	1248	4.94	4.26E-02
SLAMF1	117	749	6.42	4.27E-02
LDLRAD3	758	115	-6.58	4.34E-02
PCDHAC1	739	115	-6.41	4.35E-02
(BRPF1*)	3674	616	-5.96	4.41E-02
WDFY2	117	730	6.26	4.42E-02
NEFH	953	154	-6.20	4.44E-02
PLCH1	661	96	-6.88	4.46E-02
(BPH3)*	1903	96	-6.28	4.48E-02
CNTNAP3	641	96	-6.68	4.66E-02

B3GNTL1	156	941	6.05	4.68E-02
STX4	700	115	-6.07	4.68E-02
DICER1	117	691	5.93	4.77E-02
MMAA	97	653	6.72	4.77E-02
NAMA	894	154	-5.82	4.88E-02
C22orf26	117	691	5.93	4.89E-02
VIL1	719	115	-6.24	4.89E-02
NFRKB	622	96	-6.48	4.91E-02
DTL	97	615	6.32	4.97E-02
TSPYL5	97	634	6.52	5.00E-02

Table S8. Differentially expressed (P<0.05) transcripts in HS vs. LS samples. Genes depicted in bold type are also present in the BPH vs. LS list (Supp. Table S4). Genes depicted in red are also present in the BPH vs. HS list (Supp. Table S5).

Genes	LS	HS	Fold Change	adj. *P*-value
MTND1P23	11858	422	-28.07	7.74E-04
HSD17B4	1875	96	-19.53	9.17E-04
MPP5	1859	96	-19.37	1.02E-03
MT-ATP8	10319	461	-22.39	1.18E-03
LCOR	1783	115	-15.48	1.90E-03
MT-ND4L	3018	269	-11.23	2.04E-03
ITGAV	1463	96	-15.24	3.16E-03
BAZ1A	1463	96	-15.24	3.57E-03
ATAD5	1738	134	-12.93	3.81E-03
SMARCA4	122	1440	11.81	4.04E-03
RBPJ	1357	96	-14.13	4.11E-03
PFKL	76	1133	14.87	4.12E-03
XKR4	1707	154	-11.11	4.19E-03
WDR35	1341	96	-13.97	4.39E-03
INTU	2561	288	-8.89	4.80E-03
MTND5P7	1448	115	-12.57	6.59E-03
ASB14	1280	96	-13.33	6.71E-03
EPB41L2	2210	269	-8.22	6.81E-03
TEX9	1738	173	-10.05	6.85E-03
MYCBP2	2225	288	-7.73	8.31E-03
KIRREL	122	1210	9.92	8.34E-03
ASH1L	1509	154	-9.82	8.58E-03
CYB5R4	1448	134	-10.77	8.60E-03
FASN	107	1095	10.26	9.12E-03
CLUHP3	76	941	12.35	9.14E-03
PHLDB3	76	941	12.35	9.16E-03
SMCHD1	2393	326	-7.33	9.35E-03
FAM154A	76	903	11.84	9.84E-03
IPO8	1463	154	-9.52	9.90E-03
KCTD15	122	1191	9.76	1.00E-02
KTN1	2926	422	-6.93	1.01E-02
PHIP	2210	307	-7.19	1.02E-02
ERBB2IP	1494	154	-9.72	1.03E-02
APLP1	76	903	11.84	1.04E-02
NOTCH1	76	903	11.84	1.04E-02
CBWD6	2362	326	-7.24	1.05E-02
FLT4	183	1440	7.87	1.06E-02
TMCO5A	1265	115	-10.98	1.07E-02
DNAJB2	91	999	10.92	1.13E-02
TRAPPC2L	91	999	10.92	1.13E-02
DNM3	1265	134	-9.41	1.13E-02
PTCHD2	91	941	10.29	1.14E-02
CNTN5	1174	115	-10.19	1.15E-02
PLEKHG7	1204	115	-10.45	1.19E-02

ADAM32	1936	269	-7.20	1.21E-02
PPFIA2	1204	115	-10.45	1.25E-02
CNOT4	1143	115	-9.92	1.27E-02
ANKRD36	3231	499	-6.47	1.28E-02
NCDN	91	941	10.29	1.33E-02
MLIP-AS1	1143	115	-9.92	1.34E-02
SPTLC3	1753	230	-7.61	1.38E-02
AP3B1	1143	115	-9.92	1.41E-02
DNAJB14	1006	96	-10.48	1.42E-02
ZNF142	122	1095	8.98	1.43E-02
PBXIP1	107	1037	9.72	1.44E-02
SLC8A1	2317	365	-6.35	1.46E-02
ORC4	1357	154	-8.83	1.48E-02
PRDM1	1174	134	-8.73	1.50E-02
SLC38A3	91	903	9.87	1.51E-02
ATP6AP1L	975	96	-10.16	1.55E-02
AKAP6	1722	250	-6.90	1.56E-02
LDLR	107	903	8.46	1.57E-02
CDC14A	945	96	-9.84	1.63E-02
SMAD4	1082	115	-9.39	1.65E-02
TTC3	2561	422	-6.06	1.65E-02
FEM1B	1311	154	-8.53	1.65E-02
METTL14	1082	115	-9.39	1.69E-02
ITGA4	945	96	-9.84	1.70E-02
STXBP5	1341	154	-8.73	1.70E-02
SSX2IP	930	96	-9.68	1.78E-02
TNS3	259	1594	6.15	1.78E-02
RGS3	244	1555	6.38	1.80E-02
MRPL45P2	899	96	-9.37	1.83E-02
ABCD3	899	96	-9.37	1.84E-02
CAPN14	91	845	9.24	1.86E-02
SLC41A3	122	1018	8.35	1.87E-02
KBTBD4	76	711	9.32	1.89E-02
SYNJ1	1143	134	-8.50	1.89E-02
RANBP1	122	1037	8.50	1.90E-02
RMST	884	96	-9.21	1.90E-02
COL9A3	76	730	9.58	1.91E-02
EXOC2	1036	115	-9.00	1.91E-02
PPP6R2	107	922	8.64	1.92E-02
FGF12	1036	115	-9.00	1.92E-02
MTND6P8	1036	115	-9.00	1.93E-02
(OSBP)*	585	96	6.10	1.94E-02
KIRREL3	198	1287	6.49	1.94E-02
UBE2W	1097	134	-8.16	1.95E-02
ZNF469	183	1287	7.03	1.95E-02
ALPPL2	76	691	9.07	1.96E-02
CCSER2	899	96	-9.37	1.96E-02
PTBP3	884	96	-9.21	1.97E-02
SLC43A1	91	826	9.03	1.99E-02
RP11-435B5.5	1890	307	-6.15	2.01E-02
L1TD1	1143	134	-8.50	2.03E-02
ZNF385D	884	96	-9.21	2.04E-02
TSNAXIP1	76	711	9.32	2.05E-02
NYNRIN	213	1421	6.66	2.05E-02
LACC1	1128	134	-8.39	2.12E-02
ATRNL1	1113	134	-8.28	2.13E-02
ZNF219	137	1075	7.84	2.15E-02
WDR55	869	96	-9.05	2.15E-02
MKKS	854	96	-8.89	2.16E-02
DDX55	869	96	-9.05	2.17E-02
(FTH1*)	1844	11211	6.08	2.18E-02
TONSL	91	787	8.61	2.19E-02

ZDHHC13	1448	211	-6.85	2.20E-02
PIDD	107	883	8.28	2.20E-02
SPEG	427	2362	5.53	2.21E-02
LSG1	823	96	-8.57	2.25E-02
NNT	838	96	-8.73	2.25E-02
PLEKHH2	854	96	-8.89	2.26E-02
TDO2	808	96	-8.41	2.27E-02
ZNF529	854	96	-8.89	2.27E-02
SUSD2	107	883	8.28	2.28E-02
SETSIP	198	1402	7.07	2.32E-02
PLCH1	838	96	-8.73	2.34E-02
MED12L	1036	134	-7.71	2.39E-02
SEMA3A	1783	288	-6.19	2.42E-02
SHQ1	823	96	-8.57	2.43E-02
PKN1	107	864	8.10	2.44E-02
WDR6	107	864	8.10	2.44E-02
PXN	137	999	7.28	2.44E-02
ADRBK1	76	653	8.57	2.47E-02
MANBA	1250	173	-7.23	2.49E-02
PGAP3	76	634	8.32	2.49E-02
TGM2	107	845	7.92	2.54E-02
USP33	1143	154	-7.44	2.56E-02
PTPRN2	122	922	7.56	2.59E-02
DCTN4	930	115	-8.07	2.60E-02
KPNA1	930	115	-8.07	2.60E-02
B9D1	76	615	8.06	2.63E-02
ZNF862	274	1575	5.74	2.64E-02
HMCN1	2408	461	-5.23	2.65E-02
EMID1	107	826	7.74	2.65E-02
EDC4	76	653	8.57	2.66E-02
SAMD3	1036	134	-7.71	2.68E-02
GCSAML	793	96	-8.25	2.76E-02
WRN	1128	154	-7.34	2.77E-02
NCKAP1	2362	461	-5.13	2.77E-02
VIL1	884	115	-7.67	2.79E-02
CAMK1G	122	903	7.40	2.81E-02
C12orf4	777	96	-8.10	2.85E-02
EDNRA	777	96	-8.10	2.85E-02
XPO4	899	115	-7.80	2.85E-02
MAN2B2	213	1248	5.85	2.85E-02
RP5-1112D6.8	76	538	7.06	2.89E-02
CPNE9	107	787	7.38	2.90E-02
PRSS1	107	787	7.38	2.90E-02
EXOC5	1113	154	-7.24	2.90E-02
MAP1S	107	787	7.38	2.96E-02
POLE3	91	672	7.35	3.01E-02
POLR1C	122	864	7.09	3.02E-02
SLC25A22	76	595	7.81	3.03E-02
L1CAM	91	711	7.77	3.04E-02
LHCGR	975	134	-7.26	3.05E-02
HNRNPK	1204	173	-6.97	3.06E-02
IL7	747	96	-7.78	3.07E-02
PPHLN1	1341	211	-6.35	3.08E-02
FBXW12	76	538	7.06	3.09E-02
CACNA1H	122	864	7.09	3.09E-02
CHRNA9	76	250	3.28	3.12E-02
TRIM16L	76	250	3.28	3.12E-02
RDH13	198	1229	6.20	3.12E-02
KRT36	76	499	6.55	3.13E-02
SLC29A2	76	595	7.81	3.15E-02
BMP6	76	269	3.53	3.17E-02
RCOR1	686	96	-7.14	3.20E-02

AL592494.5	76	288	3.78	3.21E-02
CTD-2587H19.1	76	288	3.78	3.21E-02
SLC38A11	716	96	-7.46	3.21E-02
STARD13-AS	76	307	4.03	3.26E-02
RAN	716	96	-7.46	3.26E-02
MPDZ	2515	518	-4.85	3.26E-02
PIGO	137	941	6.86	3.27E-02
RP11-693J15.5	960	134	-7.14	3.28E-02
DAZAP1	137	922	6.72	3.31E-02
RP6-42F4.1	945	134	-7.03	3.34E-02
PSME4	1326	230	-5.75	3.36E-02
RP11-324H6.5	91	653	7.14	3.38E-02
SYCP1	1158	173	-6.70	3.38E-02
KCNB2	198	1191	6.01	3.39E-02
DEAF1	122	845	6.93	3.40E-02
SLC37A2	122	807	6.61	3.41E-02
AP3M1	716	96	-7.46	3.42E-02
PSMG3-AS1	76	595	7.81	3.42E-02
CD3G	808	115	-7.01	3.43E-02
ATG9B	198	1171	5.91	3.48E-02
YME1L1	930	134	-6.92	3.49E-02
GHc-362H12.3	91	595	6.51	3.52E-02
TCF12	823	115	-7.14	3.53E-02
CYP2A13	76	518	6.80	3.54E-02
FGF17	137	922	6.72	3.54E-02
LCE5A	76	480	6.30	3.55E-02
RP11-96K19.4	2241	461	-4.86	3.55E-02
PICK1	91	653	7.14	3.55E-02
USP5	183	1133	6.19	3.55E-02
MVP	107	749	7.02	3.55E-02
ADD3	1128	173	-6.53	3.56E-02
CTD-3195I5.5	76	442	5.80	3.57E-02
ZNF394	107	711	6.66	3.57E-02
RP11-143M1.3	671	96	-6.98	3.58E-02
MYSM1	1341	230	-5.82	3.59E-02
NCAM2	716	96	-7.46	3.60E-02
RP11-286B14.1	655	96	-6.83	3.60E-02
DUSP22	229	1287	5.63	3.64E-02
MTCYBP3	793	115	-6.88	3.67E-02
PDCD1	91	615	6.72	3.68E-02
PRPF4B	1311	211	-6.21	3.69E-02
NOBOX	76	422	5.54	3.69E-02
ZNF565	107	730	6.84	3.69E-02
TRPC2	122	826	6.77	3.70E-02
PRKRIR	808	115	-7.01	3.72E-02
COL18A1	137	845	6.16	3.73E-02
RSRC1	686	96	-7.14	3.75E-02
VWA7	91	653	7.14	3.76E-02
CHRAC1	76	480	6.30	3.76E-02
HSD11B1L	76	480	6.30	3.76E-02
ASS1	76	557	7.31	3.79E-02
NR1D2	808	115	-7.01	3.80E-02
SMC5	1006	154	-6.55	3.81E-02
FAR1	793	115	-6.88	3.82E-02
ALOX12B	76	518	6.80	3.88E-02
ZNF682	701	96	-7.30	3.89E-02
NKTR	2149	461	-4.66	3.90E-02
SYCP2	1097	173	-6.35	3.90E-02
PTPN18	899	134	-6.69	3.91E-02
ABCA5	1799	365	-4.93	3.92E-02
CEP97	899	134	-6.69	3.92E-02
IFT81	991	154	-6.45	3.93E-02

IREB2	899	134	-6.69	3.93E-02
KCNQ1	76	480	6.30	3.94E-02
ARID2	1296	230	-5.62	3.95E-02
PPP1R16A	122	807	6.61	3.95E-02
HOXC-AS3	76	499	6.55	3.96E-02
ANKRD18DP	625	96	-6.51	3.97E-02
RAB21	1097	173	-6.35	3.99E-02
SUCO	777	115	-6.75	4.03E-02
CDCP1	76	518	6.80	4.05E-02
NLE1	76	557	7.31	4.07E-02
SRBD1	1082	173	-6.26	4.10E-02
GOLGA7B	91	615	6.72	4.11E-02
PLCH2	76	557	7.31	4.11E-02
SLC25A36	777	115	-6.75	4.11E-02
C1orf131	107	653	6.12	4.11E-02
FAM177A1	686	96	-7.14	4.12E-02
R3HDM2	1082	173	-6.26	4.13E-02
PAN2	229	1191	5.21	4.15E-02
UBB	76	346	4.54	4.15E-02
DCBLD2	2088	461	-4.53	4.17E-02
EIF3F	1692	326	-5.18	4.17E-02
FAM225A	107	711	6.66	4.19E-02
THNSL2	107	711	6.66	4.19E-02
MAT1A	122	787	6.46	4.20E-02
MOB1B	975	154	-6.35	4.22E-02
COPB2	1311	250	-5.25	4.22E-02
VLDLR	777	115	-6.75	4.23E-02
SPCS3	777	115	-6.75	4.24E-02
AGFG1	960	154	-6.25	4.26E-02
ESRP1	716	115	-6.22	4.28E-02
CNTRL	2149	480	-4.48	4.29E-02
SLC26A6	290	1459	5.04	4.29E-02
LRRK1	274	1325	4.83	4.30E-02
CCDC88B	198	1114	5.62	4.30E-02
CLCN2	137	845	6.16	4.31E-02
RP11-7M8.2	76	442	5.80	4.31E-02
THBS2	945	154	-6.15	4.31E-02
RP11-174G6.5	640	96	-6.67	4.32E-02
NF1	2454	557	-4.41	4.33E-02
GPR98	3887	864	-4.50	4.35E-02
PRSS8	76	538	7.06	4.37E-02
IVL	76	307	4.03	4.37E-02
CCDC18	732	115	-6.35	4.39E-02
ZFYVE16	1158	211	-5.48	4.40E-02
TOX4	960	154	-6.25	4.41E-02
DPP9	274	1325	4.83	4.42E-02
SYNJ2	335	1613	4.81	4.43E-02
MAPK12	107	691	6.48	4.44E-02
C5orf64	655	96	-6.83	4.45E-02
C9orf153	701	115	-6.09	4.48E-02
TCTE3	76	288	3.78	4.50E-02
TCEA3	747	115	-6.48	4.50E-02
PGLS	76	499	6.55	4.50E-02
USP37	1280	250	-5.13	4.51E-02
DHRS2	91	595	6.51	4.55E-02
GTF3C1	320	1479	4.62	4.56E-02
ZNF835	91	518	5.67	4.58E-02
RP11-115D19.1	579	96	-6.03	4.58E-02
MITD1	930	154	-6.05	4.59E-02
UGGT2	945	154	-6.15	4.60E-02
FAM27A	91	595	6.51	4.61E-02
SRSF5	1036	173	-6.00	4.63E-02

RP11-99J16__A.2	76	269	3.53	4.66E-02
RP1-18D14.7	107	653	6.12	4.66E-02
COL11A1	1280	250	-5.13	4.66E-02
UVRAG	640	96	-6.67	4.67E-02
MFSD2B	244	1267	5.20	4.68E-02
PARVG	198	999	5.04	4.71E-02
ADAM11	107	672	6.30	4.71E-02
NUCB2	1021	173	-5.91	4.73E-02
CSK	91	595	6.51	4.74E-02
RP11-17M15.2	76	365	4.79	4.76E-02
RP11-214L19.1	76	365	4.79	4.76E-02
C4orf29	732	115	-6.35	4.76E-02
DGKH	1997	461	-4.33	4.77E-02
TEKT5	107	653	6.12	4.78E-02
ABI3BP	1951	442	-4.42	4.78E-02
CAPS2	1021	173	-5.91	4.80E-02
SSBP2	1021	173	-5.91	4.80E-02
HMGCLL1	930	154	-6.05	4.80E-02
ZNF66	625	96	-6.51	4.80E-02
NEK8	76	518	6.80	4.81E-02
MMP11	107	672	6.30	4.83E-02
GBAP1	76	480	6.30	4.83E-02
EML4	1021	173	-5.91	4.84E-02
HHLA2	1021	173	-5.91	4.84E-02
PAPD4	1280	230	-5.56	4.84E-02
CTD-2587H24.5	107	634	5.94	4.84E-02
CDH12	1235	230	-5.36	4.87E-02
C22orf26	122	691	5.67	4.87E-02
RP1-225E12.2	823	134	-6.12	4.88E-02
TIA1	823	134	-6.12	4.88E-02
DSCAML1	183	941	5.14	4.89E-02
RP4-791M13.4	579	96	-6.03	4.89E-02
FCN1	76	250	3.28	4.90E-02
NCR2	107	595	5.58	4.91E-02
GBA	107	634	5.94	4.92E-02
SUCLG2	594	96	-6.19	4.92E-02
PPP6R1	137	807	5.88	4.92E-02
SIGLEC8	198	1095	5.52	4.92E-02
VIPR1-AS1	76	480	6.30	4.93E-02
AKAP9	3033	711	-4.27	4.96E-02
NAALAD2	899	154	-5.85	4.96E-02
FLOT1	122	711	5.83	4.97E-02
VPS9D1	122	711	5.83	4.97E-02
PIAS1	549	96	-5.71	4.97E-02
PKD1	351	1517	4.33	4.97E-02
CENPV	76	346	4.54	4.98E-02
NOX3	76	346	4.54	4.98E-02
ADAM33	76	499	6.55	4.99E-02
C19orf44	76	461	6.05	5.00E-02

Table S9. Statistically significant pathways for differentially expressed transcripts in BPH and LS samples (*n*= 327).

	**-log(p-value)**
Aldosterone signaling in epithelial cells	9.94×10^{-4}
**Protein Ubiquitination Pathway**	3.35×10^{-3}
Glioma signaling	2.8×10^{-3}
iCOS signaling in T helper cells	8.52×10^{-3}
Antiproliferative role of TOB in T cell signaling	4.7×10^{-3}
PI3K signaling in B lymphocytes	2.27×10^{-2}
Neuregulin signaling	3.14×10^{-2}
Ceramide biosynthesis	4.12×10^{-2}

Serotonin and melatonin biosynthesis	4.2×10^{-2}
Glycogen biosynthesis	4.2×10^{-2}
Role of OCT4 in mammalian embryonic stem cell pluripotency	4.26×10^{-2}
Tryptophan degradation	4.89×10^{-2}

Table S10. Statistically significant pathways for differentially expressed transcripts in BPH and HS samples (*n* = 63).

	**-log(p-value)**
NER pathway	2.71×10^{-2}
Assembly of RNA I polymerase complex	2.94×10^{-2}
NAD phosphorylation and dephosphorylation	3.14×10^{-2}
**RAN signaling**	4.15×10^{-2}

Table S11. Statistically significant pathways for differentially expressed transcripts in LS and HS samples (*n* = 341).

	**-log(p-value)**
**RAN Signaling**	1.42×10^{-3}
Clathrin-mediated Endocytosis Signaling	4.87×10^{-3}
Sumoylation Pathway	1.28×10^{-2}
Axonal Guidance Signaling	1.52×10^{-2}
Paxillin Signaling	1.66×10^{-2}
Caveolar-mediated Endocytosis Signaling	1.69×10^{-2}
D-myo-inositol (1,4,5)-trisphosphate Degradation	2.15×10^{-2}
D-myo-inositol Hexakisphosphate Biosynthesis II	2.39×10^{-2}
D-myo-inositol (1,3,4)-trisphosphate Biosynthesis	2.39×10^{-2}
Palmitate Biosynthesis I (Animals)	2.67×10^{-2}
Fatty Acid Biosynthesis Initiation II	2.67×10^{-2}
**Protein Ubiquitination Pathway**	3.2×10^{-2}
Tryptophan Degradation III (Eukaryotic)	3.29×10^{-2}
D-myo-inositol (1,4,5)-trisphosphate Metabolism	3.79×10^{-2}
S-adenosyl-L-methionine Biosynthesis	3.98×10^{-2}
4-aminobutyrate Degradation I	3.98×10^{-2}
Hepatic Fibrosis / Hepatic Stellate Cell Activation	4.03×10^{-2}
Apelin Cardiomyocyte Signaling Pathway	4.61×10^{-2}
Apelin Liver Signaling Pathway	4.74×10^{-2}
RAR Activation	4.77×10^{-2}
Regulation of the Epithelial-Mesenchymal Transition Pathway	4.77×10^{-2}

Table S12. Sequences used for Taqman probe design.

OSBP (Chr 11:59,337,733-59,387,385 (NM_02556.3)), exon sequence 2171 to 2391:

CATCCTCTGTGGCTTTCATAGCTTCCGCTTCTCTCTTCTTTCTGGAAAGTCTTTGTTTTTCCTCCAGGCGC
TGCTTCTCCGCATTTGCTTCATCCCAGCGTCCATTTTCCATAGTCTCTGGTCAGGTCGTAACCGGCTGTC
TGTGGGGGCAGTGCCACTTTCCCAACATTGAGAGTCAGAGCAAGCTCTGAGAAGTAGTACATGTTTTC
TGCATTCTTC

PHC3 (Chr 3:169,795,957 - 169,908,936 (NM_024947.4)), exon sequence 938 to 1182:

CTGAATCGGTGAACAATGCTGTGACTGGGCATTACTGGGAGCTGGAGGAAGGCCATGGTTCTGGAGTG
GTATACAGTGCTGGGATGGGGGTGGGTCTTGAGTTGAATTCTGAAGTGTGATTGGCTGAATTTGCTGTT
GCTGCTGTTGTAATATCAGCTGATGATGGGAAACTTTGGAAGGTGGTGAATGAAGAGGAATCTGCTGA
TGTTTTATTAGAGAATGAGGCTGAATTGGAGAATATGAAG

UACA (Chr 15:70,935,992 - 71,066,798 (NM_018003.4), exon sequence 1642 to 2090:

CTTTCTCATTCACTTCATTTGATAATGAGCTCTTCATGTTTTCAAATTTTTCAGCTGGAATGGAAAGGGC
CAACTTCGCTGACAATTCTTTTGCCTGGCCTTCCATCTCTGTGACCTTTCTTCCTTTCTTCTCTCGCTCCA
TTTCACACATACTAAGTTCCTTCTGTAATCGCTTATTTTCTTCTATCAGCTTGCCTTCATCCCTCTTAAAC
TCTTCTACTATCATCTCATTTTGTTTGATTTGGTTTCTTAATTTCCCCACTTCTGCTGAAGCACCTTCATAT
TTTACTTTCAAGTCTTTCAACTGATCCTTCAGTTCCTCGGTTAGTCTGTGATTCCCTGAGGCTGCTTCACT
TGTTAAGTGTTCTTTAAGGGCAAGAAAATGGGTCTGCATTTGTTTAACTTTACCTTCTGACTCATACATC
CTCTTCTGCACATCTTTTAATGCACATCCTCTGTGGCTTTCATAGCTTCCGCTTCTCTCTTCTTTCTGGAA
AGTCTTTGTTTTTCCTCCAGGCGCTGCTTCTCCGCATTTGCTTCATCCCAGCGTCCATTTTCCATCAGTCT
CTGGTCAGGTCGTAACCGGCTGTCTGTGGGGGCAGTGCCACTTTCCCAAGCATTGAGAGTCAGAGCAA
GCTCTGAGAAGTAGTACATGTTTTCTGCATTCTTC

***BRPF1* (Chr 3:9,771,791 - 9,791,327 (NM_001003694.2)), exon sequence 1014 to 1973:**

GTACATCGAGAAGTCTGCAGAGGAGCTGGACGAGGAAGTAGAGTATGACATGGACGAGGAGGACTA
CATCTGGCTGGATATCATGAATGAGCGTCGGAAGACAGAGGGTGTAAGTCCCATCCCGCAGGAGATC
TTTGAGTACCTAATGGACCGACTGGAGAAGGAGTCGTACTTTGAGAGTCATAATAAAGGCGACCCTA
ATGCGCTAGTGGACGAGGATGCTGTTTGCTGTATCTGCAATGATGGTGAGTGCCAGAACAGCAATGTC
ACCTCTTCTGTGACATGTGCAACCTGGCCGTGCACCAGGAGTGCTACGGTGTCCCCTATATCCCTGAGG
GCCAGTGGCTGTGCCGCCGTTGCCTGCAGTCACCCTCTCGTGCTGTGGATTGTGCCCTGTGCCCCAACA
AGGGCGGTGCCTTCAAGCAGACAGATGACGGGCGCTGGGCCCATGTGGTGTGTGCCTTGTGGATCCCT
GAGGTCTGCTTCGCCAACACGGTCTTCCTAGAGCCTATTGACAGCATTGAGCACATCCCACCAGCTCG
CTGGAAGCTCACCTGCTACATTTGCAAACAACGGGGCTCAGGGGCCTGCATCCAGGCCACAAGGCCA
ACTGTTACACAGCTTTCCATGTGACATGCGCCCAGCAGGCTGGCCTTTACATGAAGATGGAGCCTGTG
CGGGAGACAGGCGCCAACGGCACCTCTTTCAGTGTCCGCAAGACAGCCTACTGCGACATCCACACGC
CTCCAGGTTCAGCACGCCGACTGCCTGCCCTGTCCCACAGCGAGGGTGAGGAGGATGAAGATGAGGA
GGAGGATGAGGGTAAGGGCTGGAGCTCAGAGAAAGTCAAGAAGGCCAAGGCCAAGTCCCGGATCAA
AATGAAGAAGGCACGGAAGATCCTGGCAGAGAAGCGGGCAGCAGCACCTGTGGTGTCAGTGCCCTG
CATCCCACCACACAG

***FTH1* (Chr 11:61,731,419 - 61,735,470 (AC004228.2)), exon sequence 188806 to 189645:**

TCAATCTTTGAGGCTGCAGGCAGGCACCCATCTCCCCATTTCACAGGCAGGGAAACTGAGGTCCAGAG
AGAGGGAGAGATTCCTCCAAGTCATCAGGCACATACAAGGTCCTGCCTGGGATGATCTTTCTGTGGGA
CTTCTTCTGTCCCTGGTGACCAGGTGTCCCTGTTGGCTGTGGATGAGATGCACCAGGACCTGCCTCGGA
TGGAGCCGGACATGTACTGGAATAAGCCCGAGCCACAGCCCCCCTACACAGCTGCTTCCGCCCAGTTC
CGTCGAGCCTCCTTTATGGGCTCCACCTTCAACATCAGGTGTGGCCAGAGCCAGGGGGCTGGGTGGGA
AGCCCCTCCTAGTGCAGGGGTCTGCCTAGGAACTTAGAATAGCACTAGTTAATGCATACAGGTTGCTT
CAGTAAGTGTCAGGCACTGTACTATGCTCTTTATAAACATTAACTATTTTTTTCCTCCCAATAATTCTGG
TTTGTTATCCCAAGTTTTCAGATAATTAAAGTACAGGTTCAGAGAGAGTAAGTTGTCCAAGGCCACAT
AGCTACCAAATGGTGCATTTGCTACTCGAAGGACAGCCTGTGATCAGTGATGCAGTGGAACGTTAGGA
CCTGGCTCTTGTCATCCAGAACTATGTTTTCTTTTCTTTTTGAGACAGTATCTCGCTCTGTCGCCCAGGTT
GGAGCGCAGTGGCGTGATCTTGGCTCACTGCAACCTCCGCCTCCTGGGTTCAAGTGATTCTCCTGCTTC

AGCCTCCCCAGTAGCTGGGATTACAGGTGCCCACAACCACAACTGGCTAATTTTTGTACTTTTAGTAGA GATGAGGTTTCACCAT

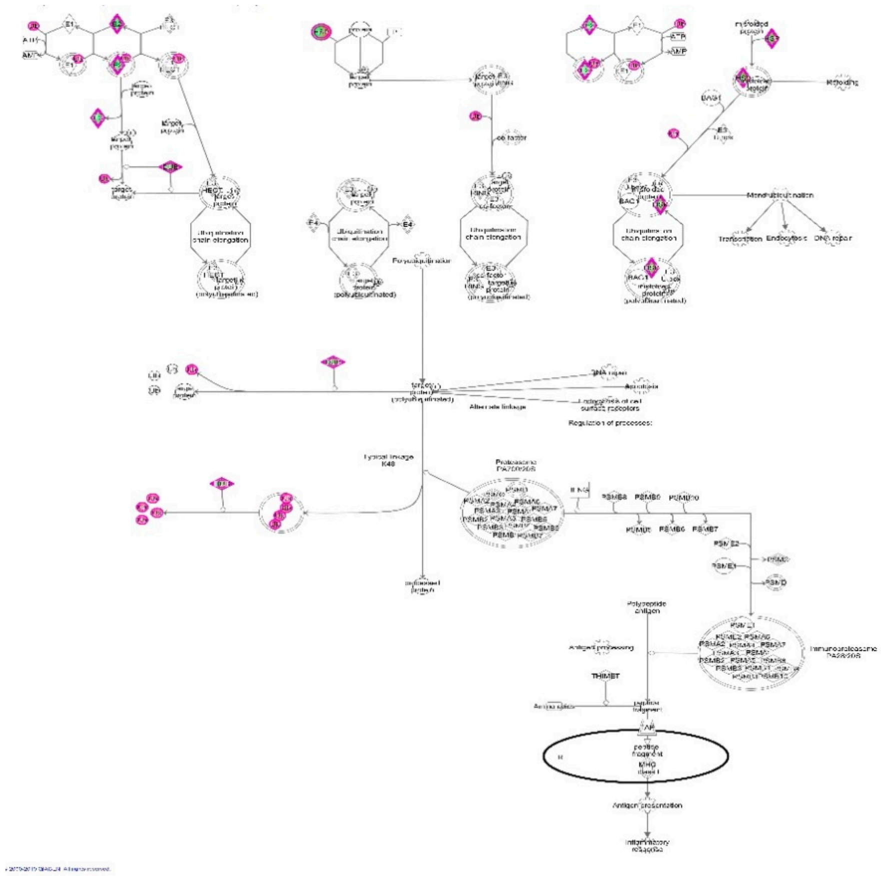

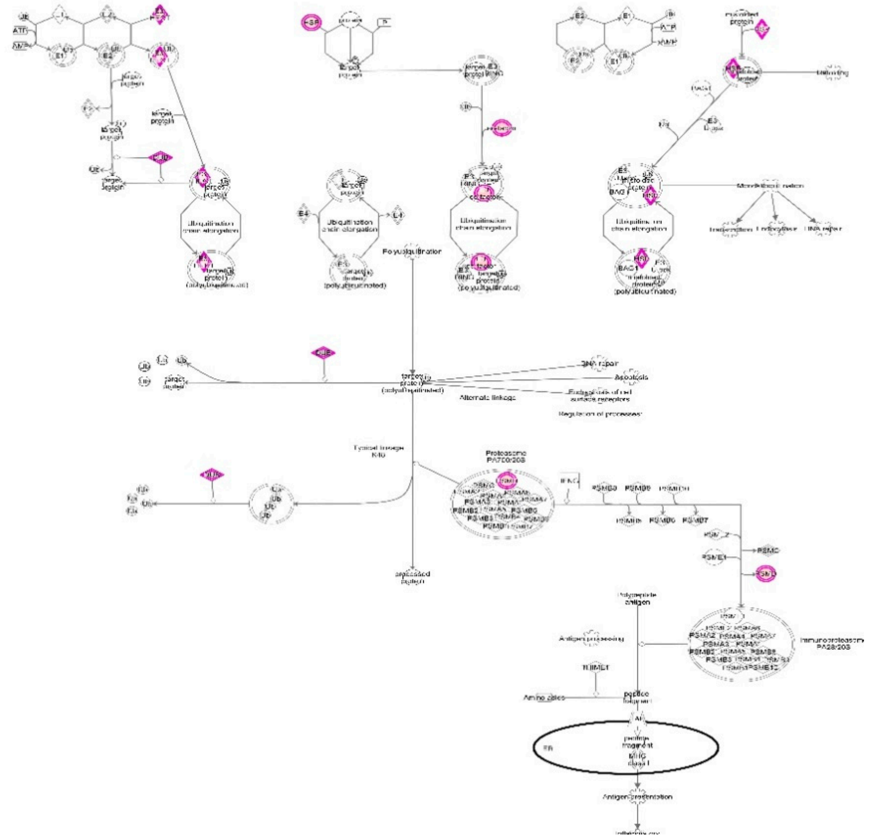

Figure S1. Differentially expressed genes in LS vs. HS (top) and BPH vs. LS (bottom) in the context of the protein ubquitination pathway. Differentially expressed genes are depicted in pink. .

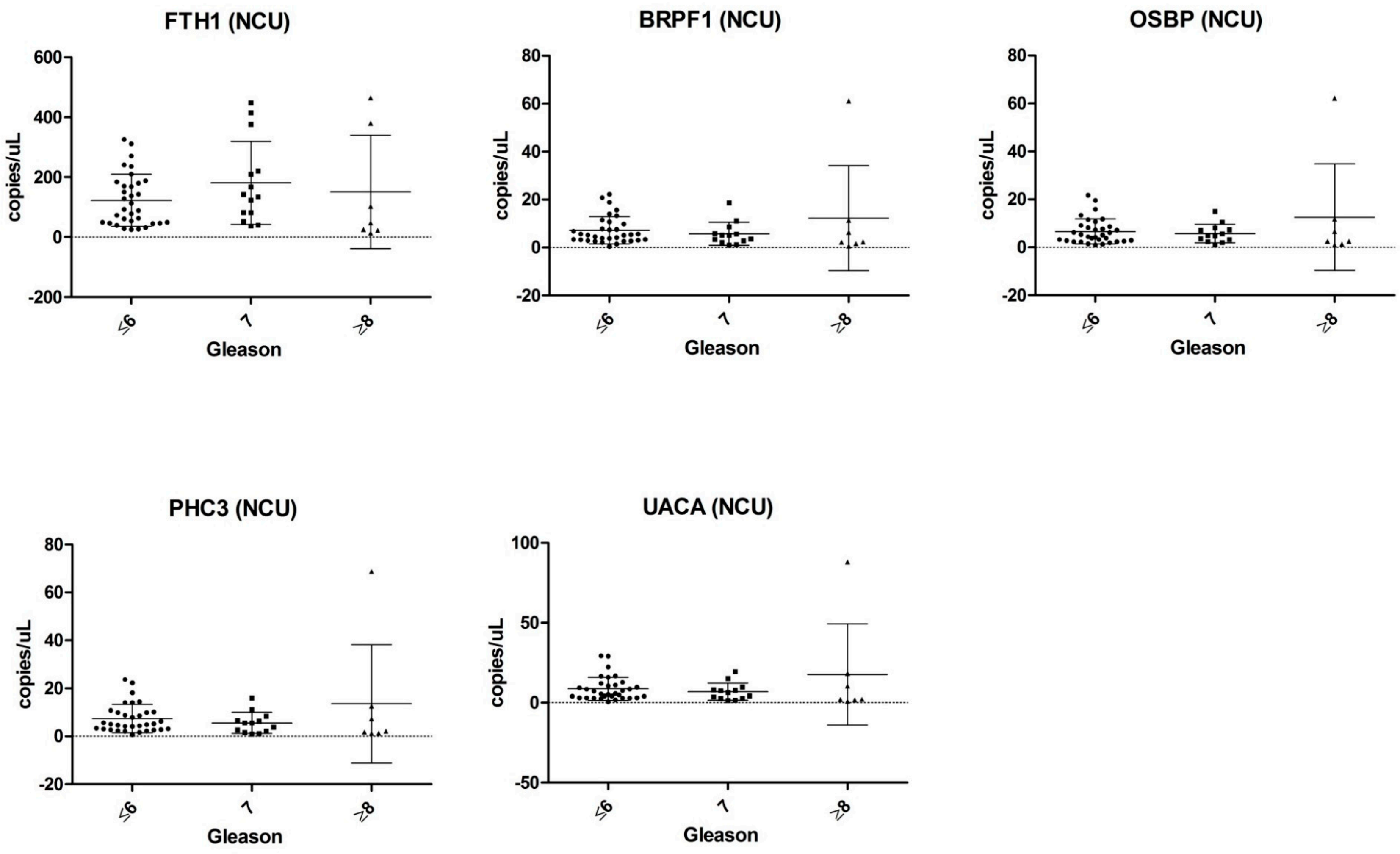

Figure S2. Expression levels of selected genes in non-centrifuged urine samples (NCU), classified according to the Gleason score. Expression levels were compared using the Mann–Whitney independent *t*-test (* $p \leq 0.05$).

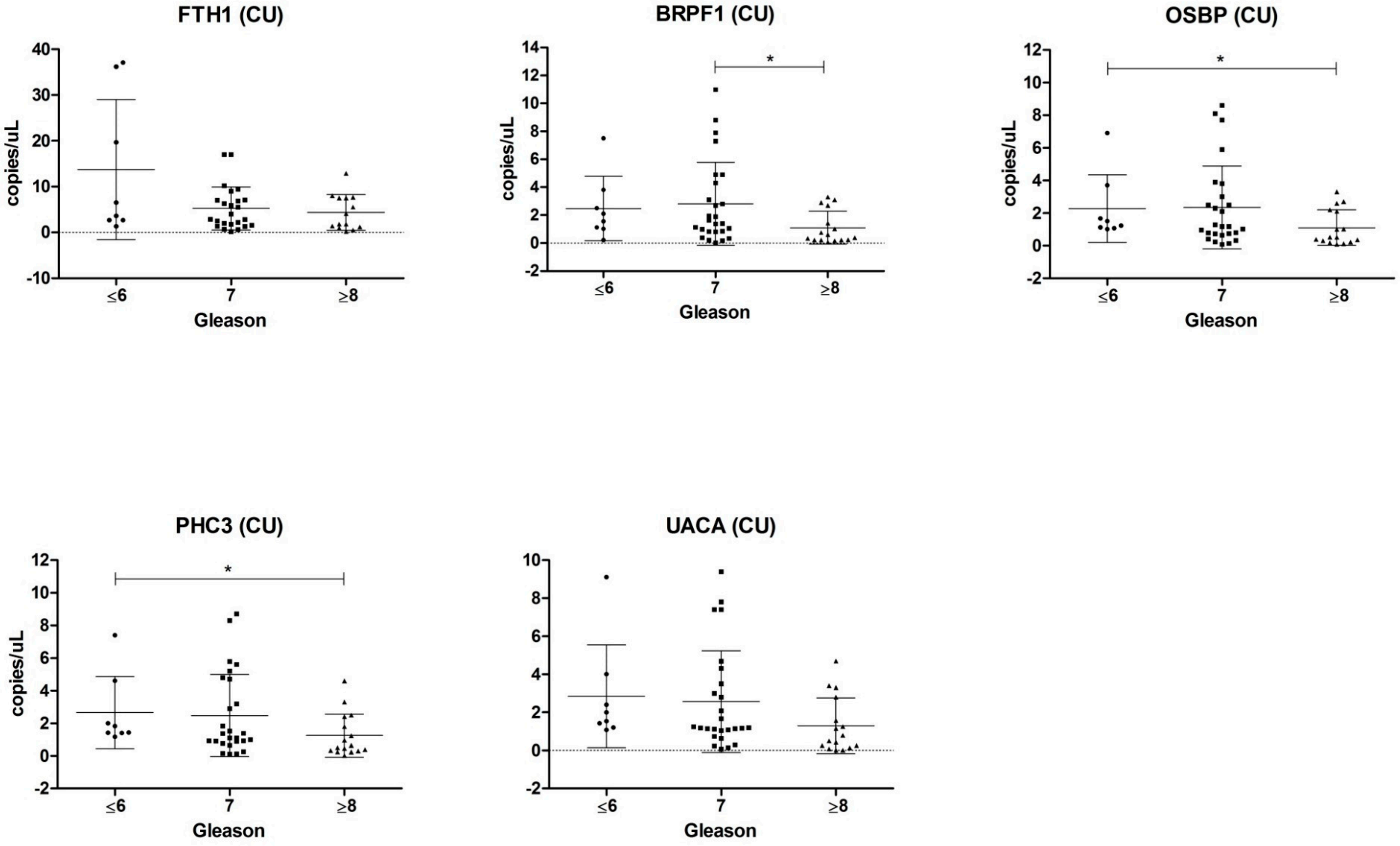

Figure S3. Expression levels of selected genes in centrifuged urine samples (CU), classified according to the Gleason score. Expression levels were compared using the Mann–Whitney independent t-test (*$p \leq 0.05$).
